# Mutualism with sea anemones triggered the adaptive radiation of clownfishes

**DOI:** 10.1186/1471-2148-12-212

**Published:** 2012-11-02

**Authors:** Glenn Litsios, Carrie A Sims, Rafael O Wüest, Peter B Pearman, Niklaus E Zimmermann, Nicolas Salamin

**Affiliations:** 1 Department of Ecology and Evolution, Biophore, University of Lausanne, 1015, Lausanne, Switzerland; 2 Swiss Institute of Bioinformatics, Génopode, Quartier Sorge, 1015, Lausanne, Switzerland; 3 Ecological and Evolutionary Genetics Lab / Coral Reef Ecology Lab, School of Biological Sciences, The University of Queensland, 4072, St Lucia, Australia; 4 Landscape Dynamics, Swiss Federal Research Institute WSL, 8903, Birmensdorf, Switzerland

**Keywords:** Ecological speciation, Diversification, Comparative method, Evolutionary rate, Brownian Motion, Pomacentridae

## Abstract

**Background:**

Adaptive radiation is the process by which a single ancestral species diversifies into many descendants adapted to exploit a wide range of habitats. The appearance of ecological opportunities, or the colonisation or adaptation to novel ecological resources, has been documented to promote adaptive radiation in many classic examples. Mutualistic interactions allow species to access resources untapped by competitors, but evidence shows that the effect of mutualism on species diversification can greatly vary among mutualistic systems. Here, we test whether the development of obligate mutualism with sea anemones allowed the clownfishes to radiate adaptively across the Indian and western Pacific oceans reef habitats.

**Results:**

We show that clownfishes morphological characters are linked with ecological niches associated with the sea anemones. This pattern is consistent with the ecological speciation hypothesis. Furthermore, the clownfishes show an increase in the rate of species diversification as well as rate of morphological evolution compared to their closest relatives without anemone mutualistic associations.

**Conclusions:**

The effect of mutualism on species diversification has only been studied in a limited number of groups. We present a case of adaptive radiation where mutualistic interaction is the likely key innovation, providing new insights into the mechanisms involved in the buildup of biodiversity. Due to a lack of barriers to dispersal, ecological speciation is rare in marine environments. Particular life-history characteristics of clownfishes likely reinforced reproductive isolation between populations, allowing rapid species diversification.

## Background

The concept of adaptive radiation has been central to evolutionary biology since Darwin’s work on Galapagos finches [1-3]. The general understanding of this process is that rates of ecomorphological changes and species diversification will be increased by ecological opportunities offering available resources untapped by competing species [4]. Ecological opportunity can arise for four main reasons [5], the most widely described being the colonisation of geographically isolated areas with depauperate fauna (e.g. cichlid fishes in East-African Great Lakes [6]). The process is similar in the aftermath of a mass extinction event, which allows surviving species to radiate rapidly by filling the available empty niches [7]. Modification of a resource can also trigger native species radiation as demonstrated by the radiation of *Lupinus* in high-elevation habitats that appeared during the Andean uplift [8]. Finally, the appearance of a trait allowing new interactions with the environment, or key innovation, can create an opportunity for species radiation [9]. For example, the evolution of antifreeze glycoproteins found in notothenioid fishes of Antarctica is thought to have triggered their adaptive radiation by allowing survival in extreme environments [10]. In an analogous manner to key innovations, the evolution of mutualistic interactions can provide access to previously inaccessible resources. For instance, phytophagous insects host mutualistic microbes, which enable the breakdown and digestion of plant compounds by the insects [11]. While a plethora of case studies showing adaptive radiation driven by ecological opportunity offered by one of the aforementioned possibilities exist [9], examples involving mutualism are scarce (but see [12]). Since hosts shifts have allowed ecological speciation in a wide range of organisms, including coral-dwelling fish [13,14], there is a possibility for ecological speciation to occur in mutualistic systems. However, results from empirical and theoretic studies give contradictory evidence on the effect of mutualism on species diversification [15,16]. The topic is thus still debated and in need of further case studies.

**Figure 1:**
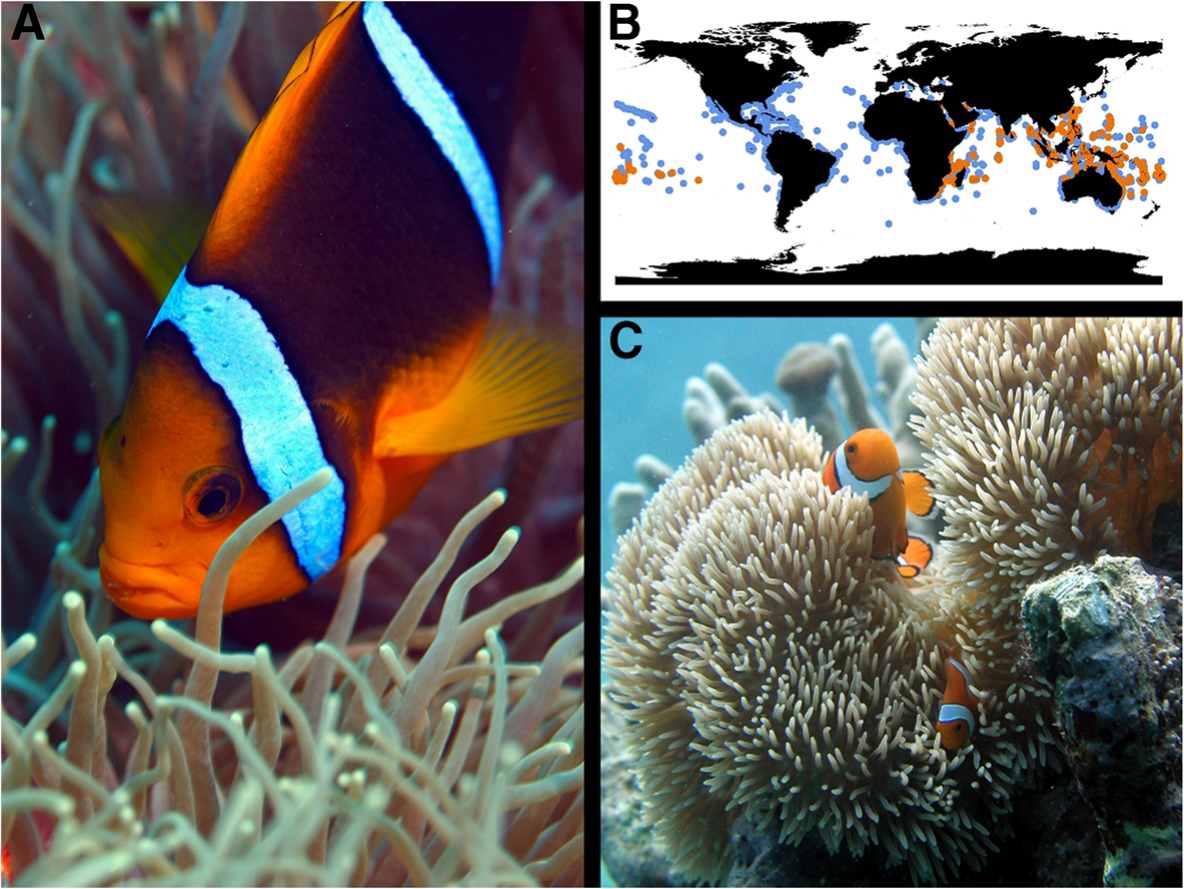
***Clownfishes and sea anemones mutualism, and geographic distribution. *** Illustration of the mutualistic relationship between *Amphiprion chrysopterus* and *Heteractis crispa***(A)**. The distribution of the damselfishes in blue and of the clownfishes in orange is shown in panel **B**. As for every clownfish species, the female *Amphiprion percula* (on top of the picture of panel **C**, here with *Stichodactyla gigantea*) is bigger than the male beneath.

The clownfishes (or anemonefishes; subfamily Amphiprioninae) are a group of 30 species within the damselfish family (Teleostei; Perciformes; Pomacentridae; [17]) and are emblematic species of coral reefs (Figure 1A &1C). Their distribution spans from the Indian to the western Pacific Oceans (Figure 1B) with their highest species richness found in the Indo-Malay archipelago where up to nine species have been observed in sympatry [18]. Their complex association with sea anemones is now a textbook example for mutualistic interactions [19-21]. Clownfishes are left unharmed by the otherwise lethal nematocysts of the sea anemone tentacles. This ability is thought to come from a protective mucous coat that prevents the discharge of the nematocysts [22] and allows clownfishes to settle in sea anemones. The protection against predators provided by the sea anemones is a direct advantage for clownfishes. Likewise, clownfishes chase the predators of the sea anemones. Furthermore, waste ammonia excreted by the clownfishes is used by the endosymbiotic dinoflagellates living in the sea anemone tissues, which makes it a three-way interaction [19,21]. The efficiency of the protection provided by the sea anemone is demonstrated by the extraordinary life span of clownfishes (ca. 30 years recorded for *Amphiprion percula*), which is twice as long as any other damselfish and six times greater than the expected longevity for a fish of that size [23].

While species of clownfishes can develop mutualistic interactions with up to ten species of sea anemones (Table 1), a large variation in host usage exists within the clade [20]. Eight host sea anemones have a widespread distribution and two (*Heteractis malu* and *Macrodactyla doreensis*) have more restricted ranges, but are distributed around the centre of diversity for the clownfishes, making interaction between most clownfishes and host species geographically possible [20]. Although geographically widespread, sea anemone species differ in their preferred habitat (e.g. reef zonation, substrate, depth; [24]). It was shown that coexistence of multiple clownfish species was possible because of difference in host and habitat utilisation [18]. It is therefore possible that the appearance of mutualism was the key innovation that allowed the clownfishes to diversify in ecological niches associated with the different sea anemones species. However, this hypothesis has never been tested thoroughly.

**Table 1:** Interaction matrix between clownfishes and their sea anemone hosts

	***Cryptodendrum adhaesivum***	***Entacmaea quadricolor***	***Heteractis aurora***	***Heteractis. crispa***	***Heteractis magnifica***	***Macrodactyla doreensis***	***Stichodactyla gigantea***	***Stichodactyla haddoni***	***Stichodactyla mertensii***	***Heteractis malu***
*Amphiprion akallopisos*					+				+	
*Amphiprion akindynos*		+	+	+	+			+	+	
*Amphiprion allardi*		+	+						+	
*Amphiprion barberi*		+		+						
*Amphiprion bicinctus*		+	+	+	+		+		+	
*Amphiprion chagosensis*		(+)								
*Amphiprion chrysogaster*			+		+			+	+	
*Amphiprion chrysopterus*		+	+	+	+	+		+	+	
*Amphiprion clarkii*	+	+	+	+	+	+	+	+	+	+
*Amphiprion ephippium*		+		+						
*Amphiprion frenatus*		+								
*Amphiprion fuscocaudatus*									+	
*Amphiprion latezonatus*				+						
*Amphiprion latifasciatus*									+	
*Amphiprion leucokranos*				+	+				+	
*Amphiprion mccullochi*		+								
*Amphiprion melanopus*		+		+	+					
*Amphiprion nigripes*					+					
*Amphiprion ocellaris*					+		+		+	
*Amphiprion omanensis*		+		+				+		
*Amphiprion pacificus*					+					
*Amphiprion percula*				+	+		+			
*Amphiprion perideraion*				+	+	+	+			
*Amphiprion polymnus*				+		+		+		
*Amphiprion rubrocinctus*		+					+			
*Amphiprion sandaracinos*				+					+	
*Amphiprion sebae*								+		
*Amphiprion thiellei*				(+)					(+)	
*Amphiprion tricinctus*		+	+	+					+	
*Premnas biaculeatus*		+								

Known interactions are shown by plus signs. Field records are lacking for *A. chagonsensis* and *A. thiellei*, the most probable host is shown between parentheses. The species status of *A. leucokranos* and *A. thiellei* is debated as they may be natural hybrids [20].

Examples of ecological speciation events are rare in marine ecosystems. This is likely due to the fact that long-distance dispersal is common among marine organism [25]. However, clownfishes are known to have high larval retention to natal reefs [26,27]. They are also known to produce species-specific calls [28,29] that differ among geographic populations [30]. Such properties are likely to have reinforced reproductive isolation by reducing gene-flow between clownfishes populations and thus facilitated ecological speciation processes in clownfishes.

In this study, we test whether the evolution of the mutualism with sea anemone in the clownfishes lineage is a key innovation that led to ecological adaptive radiation (sensu [4]). We ensure that the clownfishes are monophyletic by building a phylogeny for the Pomacentridae family. Next, we show the occurrence of rapid speciation in the clownfishes by testing whether their diversification rate is higher than that of the other damselfishes. We then use an ordination method on the mutualistic interactions to describe potential ecological niches associated with the sea anemones. We further apply phylogenetic comparative methods to test the association between morphological traits and the putative ecological niches. We finally measure the rate of evolution of the morphological traits to see if they fit the theoretical expectation of faster morphological evolutionary rate [4,31].

## Methods

### Mutualism and clownfishes phenotype

We collected data on the distribution of clownfishes among the 10 possible sea anemone host species (Table 1; [19,20,32]). We applied a multiple correspondence analysis (MCA) on the matrix of mutualistic interactions between sea anemones and clownfishes. The MCA analysis is the counterpart of principal component analysis for categorical data, which shows the underlying structure present in the dataset. We used the axes of the MCA that explained most of the variance in the subsequent analysis. This allowed us to determine in a multivariate space, the characteristics of the ecological niches used by the clownfishes and provided by mutualistic interactions.

We extracted morphological measurements of the damselfish from the literature (mainly from [33], other sources are listed in the Additional file 1). It is thought that adaptation to sea anemones required modifications of the general shape as well as a change in swimming ability in host specialised clownfish. Indeed, continuous and fast swimming is not needed anymore because specialised species never venture far from their host [34]. We thus collected traits in the literature for all Pomacentridae species present in our phylogeny that are linked with body shape and swimming abilities as well as trophic niche, which is generally linked with habitat in Pomacentridae [35]. This analysis resulted in a matrix of eight morphological traits (maximum standard length, the ratio between standard length and the greatest body depth or “body ratio”, the count of hard and soft dorsal-fin rays, the count of soft anal-fin rays, the count of pectoral-fin rays, the number of gill rakers present on the first gill arch and the number of scales which possess a sensory tube or “lateral-line scales”). Standard length and body ratio describe the overall fish shape, which has been shown to be linked with adaptation towards habitats with differing water velocity regimes [36,37]. Fin morphology directly influences fish locomotory ability [38] and gill rakers are used as a proxy for the differentiation along the pelagic-benthic trophic resource axis [39]. The number of lateral-line scales is one of the more pronounced morphological differences between the clownfishes and other damselfishes [40], and may be of importance in the ecological adaptive radiation. It was not possible to take into account intra-specific variation in our analysis and we recorded a single value per trait estimated as the mean of the values obtained from the literature. To diminish potential allometric effects, all traits were log transformed before further analysis.

### Phylogeny and divergence time estimation

We assembled DNA sequence data for 196 Pomacentridae species (170/356 damselfishes, 26/30 clownfishes) spanning all genera in the family (Accession numbers available in Additional file 2). Three cichlid species (*Aequidens rivulatus*, *Thorichthys meeki*, *Tomocichla sieboldii*) were included as outgroups [41,42]. The concatenated sequence matrix was 6945 bp long and composed of six mitochondrial and three nuclear gene regions (12S, 16S, ATP6-8, COI, cytochrome b, ND3, BMP-4, RAG1 & RAG2). Each DNA region was aligned separately with MUSCLE [43] and ambiguously aligned nucleotides were removed using Gblocks [44].

After visually checking the alignment, we used BEAST [45] to simultaneously infer the phylogeny and estimate divergence times. We used a relaxed clock model, drawing substitution rates from a lognormal distribution. We partitioned the alignment by gene as it outperformed an unpartitioned analysis in Bayes factors in a similar dataset [41]. We selected, using Akaike information criterion values (AIC), the substitution model that fits best each partition with the function “phymltest” available in the Ape package [46] in R [47] (see the model choice in Additional file 3). We used the only fossil calibration point available for the basal node of the Pomacentridae to obtain absolute divergence time estimates. The fossil that is the earliest record of Pomacentridae (Monte Bolca, Italy) dates back to 50 million years (MY) [48], which we used as minimum age with a lognormal prior (mean = 2; sd = 1.2; prior 5-95% = 51.03-103.2) following [41]. We selected a lognormal prior to allow the basal node of the Pomacentridae to reach back to ~105 MY, which is the probable age of the Perciformes [49,50]. We performed two parallel BEAST runs, each 5*10^7^ generations long and sampled posterior distributions every 1,000 generations. We checked the convergence of the two chains, optimal sampling of model parameters and estimated the burn-in length in Tracer [45]. After the removal of 10,000 trees as burn-in, we merged both runs and inferred a maximum credibility phylogeny using TreeAnnotator [45]. Finally, we resampled from the posterior distribution 100 trees to be used in further analysis. These time calibrated trees are hereafter referred to as the distribution of chronograms. As BEAST also outputs phylograms having branch lengths given in expected number of substitution per site, we applied the same resampling procedure to get a random sample of 100 phylograms. This allowed us to test our hypothesis on two sets of phylogenies instead of possibly biasing our results by choosing arbitrarily a specific branch length unit [51].

### Diversification rate

We used the package Diversitree [52] in R to test whether mutualism with sea anemones is linked with an increased diversification rate in the clownfishes as would be expected under the key innovation hypothesis. We applied the BiSSE method [53], which evaluates jointly the evolution of a binary character (here presence or absence of mutualism with sea anemones), speciation and extinction rates. As we do not have a complete sampling of the Pomacentridae, we used an extension of the method that deals with incompletely sampled phylogenies [52]. A one-rate birth-death model is fitted to the whole tree and compared, using AIC and Likelihood ratio test, with an alternative model allowing two separate rates of speciation and extinction for clownfishes and damselfishes species. In this particular case, the clownfishes are a monophyletic group nested within the Pomacentridae phylogeny. No known clownfishes species has lost the mutualistic behaviour and we therefore forced the loss of mutualism in the model (parameter q10) to a fixed null value. We optimised the other parameters of the model (rates of speciation, extinction and probability of character change) by Maximum Likelihood estimation independently on each of the 100 randomly sampled chronograms to account for phylogenetic uncertainty. The rate of diversification was calculated by subtracting the extinction rate from the speciation rate.

### Phylogenetic signal and phenotype-environment correlation

We estimated the phylogenetic signal in the morphological data on each of the 100 phylograms and chronograms with the K [54] and λ [55] indexes as implemented in the Phytools package [56] in R. Assessing the phylogenetic signal of a trait on both phylograms and chronograms can help choose which branch length unit will be the most appropriate for comparative analysis [51]. For both indexes, a value close to 0 is diagnostic of a weak or nonexistent phylogenetic structure, while values close to one are expected if the data follows a Brownian motion (BM) model of character evolution. We performed randomisation tests for the K and a likelihood ratio test for λ to test for an observed phylogenetic signal significantly greater than 0. We repeated the analysis by taking into account only the clownfishes and this time also measuring the phylogenetic signal of the four first axes of the host usage MCA.

Past competition creating character displacement between related species will result in phenotypes that are correlated with resource usage [4]. We assessed whether the morphological traits collected are linked to host usage in the clownfish by measuring the correlation between each of the first four axes of the MCA and the eight morphological traits. We used phylogenetic generalised least squares (pGLS) as implemented in the caper package in R [57]. The λ parameter, which models the phylogenetic dependency of species trait values [55] was estimated by Maximum Likelihood and the model was replicated over each tree present in the samples of phylograms and chronograms. We assessed if the morphological variables explained a significant part of the variance in the model by running an ANOVA on the pGLS output.

### Morphological evolutionary rate

We measured the differences in rate of morphological evolution between clownfishes and damselfishes by comparing the fit of a single rate BM model to that of a multiple rate model. It has been shown that other models could better fit the data than BM especially in adaptive radiations [58,59]. We choose to use BM because our goal is solely to compare the relative rate of evolution between groups and not the actual trait values. The single rate model assumes that all lineages accumulate the same amount of morphological variance per unit of time while the multiple model allows clownfishes to have a different rate of evolution than the damselfishes. Both models were specified in the Phytools package [56] that implements the non-censored version of a typical BROWNIE analysis [60]. The best fitting model was selected according to sample size corrected AIC (AICc). We analysed each of the recorded morphological traits on the two sets of 100 trees randomly sampled from the posterior distributions of phylograms and chronograms.

## Results

### Phylogenetic inference and divergence time

**Figure 2:**
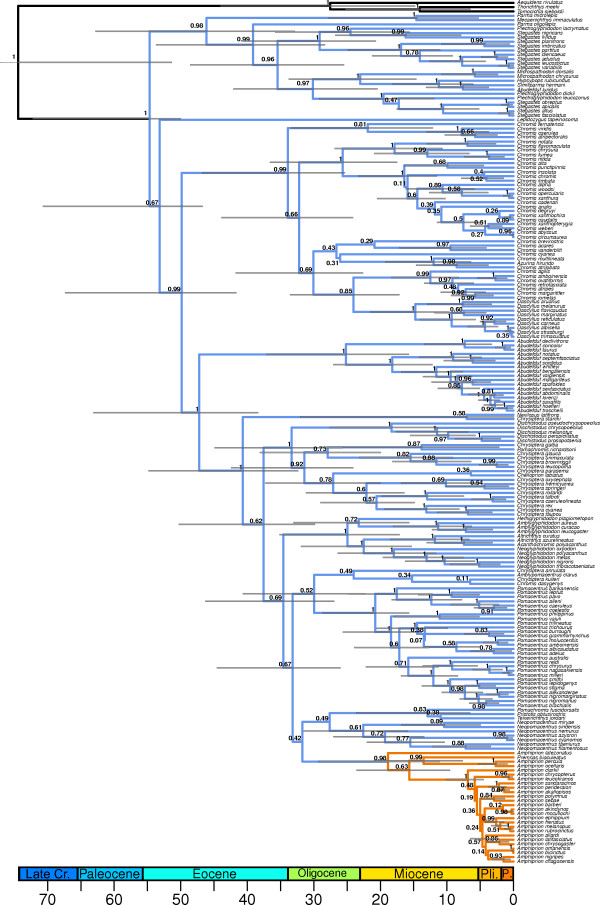
***Pomacentridae maximum credibility chronogram. *** Outgroup taxa are shown in black, damselfishes in blue and clownfishes in orange. Error bars on node show the dating confidence intervals, scale is in MY. Numbers above nodes indicate Bayesian posterior probabilities.

Our maximum credibility phylogenetic tree shows strong support for the monophyly of the clownfishes with a high posterior probability (PP = 0.98) for the basal node of the clade (Figure 2). The monophyly of the clownfishes as well as the general tree topology that we recovered was congruent with previous phylogenetic trees of the Pomacentridae [17,41,61]. Most of the nodes of the tree were highly supported (PP>0.95, Figure 2 and Additional file 4).

The estimated age of the root node of the Pomacentridae (~55 MY) was similar to previous findings [41]. We inferred a root age of ~19 MY for the clownfish clade and found the crown *Amphiprion* group, which holds most of the clownfishes species (25 out of 30 species), to have an age of ~7 MY (Figure 2).We did not include in this divergence time analysis the closure of the Isthmus of Panama. This calibration point, which constrains the split of the pairs *Abudefduf concolor/taurus* and *Abudefduf troschelli/saxatilis*[41] was not used, because biogeographic information can be uncertain when constraining the age of species splits [62,63]. However, we recovered similar dates and confidence intervals as estimated in [41].

### Speciation rate

**Figure 3:**
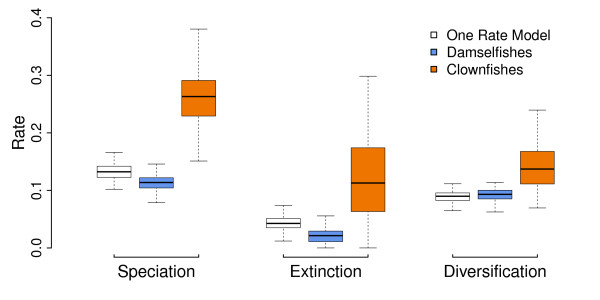
***Diversification analysis. *** Rates of speciation, extinction and diversification measured on the sample of 100 chronograms. Rates of damselfishes (mean diversification rate = 0.09) are shown in blue and clownfishes (mean diversification rate = 0.14) in orange.

We tested whether the evolution of the obligate mutualism with sea anemones fitted the expectation of a key innovation and was linked with an increased rate of speciation in the clownfish. We observed that mutualism with sea anemones was linked with higher speciation, extinction and diversification rates (Figure 3). The model allowing distinct rates of speciation and extinction for clownfishes and damselfishes also explained the data significantly better than the simpler model where both groups have the same rate (median likelihood ratio test P = 0.02). The dating and phylogenetic uncertainty are taken into account in the final result (Figure 3) because we ran these analyses on a random sample of 100 chronograms.

### Ecological niche of host usage

**Figure 4:**
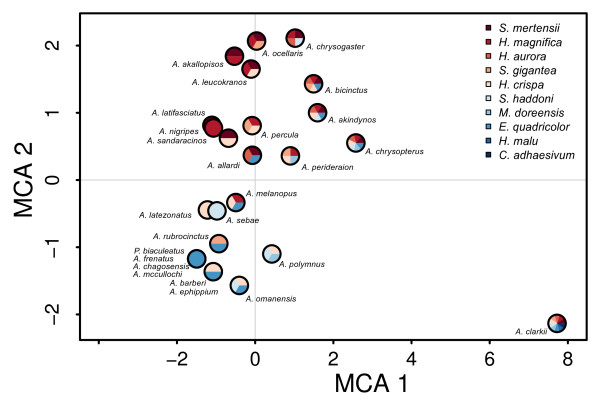
***MCA of mutualistic interactions (axes 1 and 2). *** Each pie represent a clownfish species and the filling colours correspond to the interacting sea anemone species (see legend in figure). Abbreviations: *Amphiprion*: A, *Premnas*: P, *Stichodactyla*: S, *Entacmaea*: E, *Macrodactyla*: M, *Heteractis*: H, *Cryptodendrum*: C.

**Figure 5:**
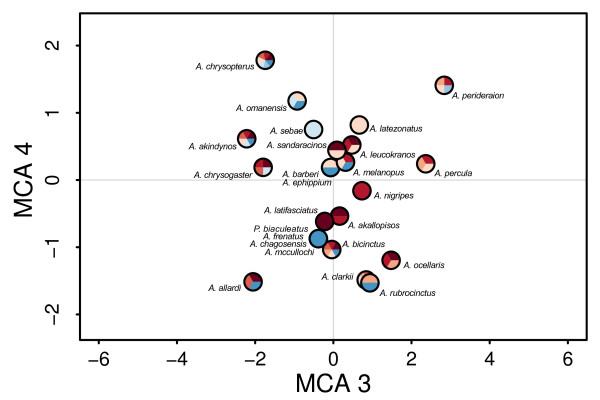
***MCA of mutualistic interactions (axes 3 and 4). *** Legend as in Figure 4.

The first four axes of the MCA explained 76% of the total variance in mutualistic interactions among clownfish species and were kept for the following analysis (Figures 4 and 5, see Additional files 5 and 6 for factorial maps). Using knowledge on sea anemone habitat and ecology [24], we could interpret the principal axes of the MCA. The first axis (35% of variance) showed a gradient of differing host usage by segregating generalists clownfishes (positive values) that have interactions with many sea anemone species from specialists (negative values), which have a small range of possible sea anemone hosts. The remaining axes showed gradients linked with habitat utilisation. Indeed, the second axis (15% of variance) separates clownfishes species interacting with sea anemones that live on different types of substrate (e.g. *Heteractis aurora* on sand and *Entacmaea quadricolor* on rock). The third axis (14% of variance) shows principally a depth gradient and the fourth axis (12% of variance) exhibits a gradient between sand dwelling sea anemone species living either among or away from coral reefs. Although this has not been formally tested, species that are close in the MCA (Figures 4 and 5) and thus similar in host usage, seldom co-occur in the wild.

### Phylogenetic signal

In the whole Pomacentridae family, the morphological traits were generally highly conserved (K and λ close to 1, Table 2). On average, the phylogenetic signal was closer to 1 when measured on phylograms than on chronograms. This would suggest that, for comparative phylogenetic methods that apply the BM model of character evolution, using phylograms would give more accurate results [51]. We nevertheless ran all the subsequent analysis on both kinds of phylogenetic trees because there is only a slight difference in the measured phylogenetic signal of our data between the phylograms and chronograms.

**Table 2:** Phylogenetic signal in the damselfishes

	**Phylograms**		**Chronograms**	
	**K**	**λ**	**K**	**λ**
Standard length	**0.407±0.11**	**0.934±0.09**	0.358±0.10	0.883±0.04
Body ratio	0.205±0.08	**0.954±0.07**	**0.233±0.09**	0.921±0.02
Dorsal fin soft rays	**0.467±0.14**	**0.925±0.06**	0.461±0.14	0.886±0.02
Dorsal fin hard rays	0.513±0.10	**0.914±0.07**	**0.578±0.12**	0.859±0.02
Anal fin soft rays	0.161±0.07	**0.911±0.07**	**0.186±0.08**	0.872±0.03
Lateral line scales	0.856±0.30	**1.026±0.06**	**0.973±0.33**	0.962±0.02
Pectoral fin rays	**0.349±0.12**	0.842±0.06	0.329±0.12	0.842±0.04
Gill rakers	0.237±0.09	**1.011±0.07**	**0.294±0.12**	0.949±0.02

Blomberg’s K and Pagel’s λ statistics of phylogenetic signal and standard deviation for the damselfish morphological dataset in the posterior distributions of phylograms and chronograms. For each trait, the statistic closest to one is indicated in bold.

In contrast to the whole Pomacentridae phylogeny, the phylogenetic signal of the morphological traits and the first four axes of the host usage MCA measured only on the clownfish clade were relatively weak (K and λ close to 0; Table 2). Only one morphological character had K value significantly different from 0 (three for the λ) while no MCA axis showed this pattern (Table 3).

**Table 3:** Phylogenetic signal of morphology and MCA axes

	**Phylograms**		**Chronograms**	
	**K**	**λ**	**K**	**λ**
Standard length	0.186±0.06	0.578±0.07	0.186±0.08	0.647±0.06
Body ratio	0.149±0.09	0.868±0.16	0.147±0.10	0.829±0.08 *
Dorsal fin hard rays	0.201±0.04	0.131±0.24	0.179±0.05	0.085±0.20
Dorsal fin soft rays	0.169±0.07	0.175±0.30	0.150±0.07	0.141±0.26
Anal fin soft rays	0.11±0.09	0.941±0.14 **	0.109±0.10	0.863±0.03 **
Lateral line scales	0.245±0.10	0.686±0.14	0.216±0.09	0.666±0.21
Pectoral fin rays	0.245±0.13 *	0.977±0.15 **	0.239±0.14	0.89±0.08 **
Gill rakers	0.068±0.05	0.721±0.11 *	0.068±0.06	0.738±0.05 **
MCA 1	0.097±0.06	0.322±0.41	0.088±0.05	0.326±0.40
MCA 2	0.033±0.03	0±0	0.032±0.03	0±0
MCA 3	0.108±0.05	0.172±0.12	0.106±0.05	0.285±0.12
MCA 4	0.04±0.03	0.021±0.15	0.038±0.03	0±0

Blomberg’s K and Pagel’s λ statistics of phylogenetic signal for the clownfish morphological dataset and host usage MCA in the posterior distributions of phylograms and chronograms. Phylogenetic signal significantly different than 0 is signified by asterisk (* = P-values <0.05, ** = P-values <0.01). Significance was assessed with a randomisation test for the K and likelihood ratio test for λ.

### Phenotype-environment correlation

We assessed the correlation between clownfish morphological traits and putative ecological niches (as described by the MCA axes) with a pGLS. The results for the models were congruent between analysis of phylograms and chronograms (Table 4). The analyses using phylograms found that all traits but body ratio and lateral line scales had a significant relation with the first axis of the MCA. Only standard length was important when correlated with the third axis of the MCA. Results were similar for chronograms, except for the pectoral-fin and hard dorsal-fin rays counts that did not significantly explain variation in the first axis of the MCA (Table 4).

**Table 4:** Correlation between morphological traits and MCA axes

		**Phylograms**		**Chronograms**	
	**Morphological trait**	**Coefficients**	**Error**	**Coefficients**	**Error**
MCA 1	Standard length	**0.264±0.321**	**1.785±0.115**	**−0.093±0.755**	**2.188±0.408**
	Body ratio	0.039±1.765	4.480±0.468	−0.036±1.435	5.618±1.423
	Dorsal fin hard rays	**8.617±1.941**	**11.096±0.715**	3.661±4.792	11.074±2.168
	Dorsal fin soft rays	**−6.904±2.517**	**12.666±0.820**	**−9.241±3.278**	**12.270±1.496**
	Anal fin soft ray	**0.155±2.183**	**9.593±0.811**	**−2.716±4.671**	**11.252±3.342**
	Pectoral rays	**−7.830±3.415**	**6.951±1.247**	−4.235±5.400	8.226±2.055
	Lateral-line scales	−4.353±1.593	6.168±0.349	−1.190±4.424	7.030±-1.230
	Gill rakers	**16.496±1.708**	**5.591±0.443**	**15.797±2.008**	**7.761±2.955**
MCA 2	Standard length	−0.920±0.085	1.452±0.013	−1.012±0.057	1.473±0.031
	Body ratio	3.505±0.143	3.950±0.054	3.781±0.459	4.029±0.024
	Dorsal fin hard rays	3.751±0.518	7.569±0.115	2.811±0.488	7.484±0.325
	Dorsal fin soft rays	10.376±0.431	7.626±0.095	9.263±0.831	7.554±0.511
	Anal fin soft ray	**−16.490±0.543**	**8.321±0.074**	**−16.398±0.890**	**8.433±0.060**
	Pectoral rays	7.134±0.241	5.685±0.060	7.356±0.747	5.742±0.003
	Lateral-line scales	0.024±0.295	4.724±0.080	0.253±1.082	4.663±0.078
	Gill rakers	4.083±0.344	6.23±-0.080	4.429±1.397	6.274±0.189
MCA 3	Standard length	**−2.421±0.097**	**1.233±0.017**	**−2.439±0.179**	**1.227±0.013**
	Body ratio	1.134±0.157	3.356±0.072	1.024±0.206	3.355±0.010
	Dorsal fin hard rays	−1.219±0.472	6.430±0.134	−1.458±0.415	6.231±0.194
	Dorsal fin soft rays	−1.975±0.435	6.479±0.131	−2.173±0.112	6.288±0.336
	Anal fin soft ray	−1.51±-0.480	7.069±0.126	−1.429±1.638	7.023±0.014
	Pectoral rays	−5.235±0.191	4.829±0.090	−5.158±0.877	4.782±0.043
	Lateral-line scales	−0.05±-0.300	4.013±0.091	0.088±1.333	3.883±0.026
	Gill rakers	−1.585±0.452	5.293±0.093	−1.566±0.030	5.227±0.189
MCA 4	Standard length	1.498±0.035	1.247±0.015	1.56±-0.060	1.248±0.010
	Body ratio	**9.968±0.191**	**3.393±0.032**	**9.783±0.670**	**3.413±0.018**
	Dorsal fin hard rays	7.273±0.567	6.502±0.097	7.820±0.112	6.337±0.180
	Dorsal fin soft rays	0.651±0.609	6.551±0.073	1.395±0.400	6.395±0.321
	Anal fin soft ray	9.599±0.288	7.148±0.065	9.327±0.908	7.142±0.030
	Pectoral rays	−2.244±0.161	4.883±0.044	−2.427±1.107	4.864±0.054
	Lateral-line scales	**−10.471±0.154**	**4.058±0.083**	**−10.593±0.200**	**3.949±0.018**
	Gill rakers	−0.620±0.151	5.352±0.067	−0.742±1.517	5.316±0.199

The table shows results and standard deviation of pGLS. Results in bold indicate variables explaining significant variation in the dependent variable as shown by the ANOVA on the pGLS output. Median adjusted R^2^ of the models on phylograms, MCA 1 = 0.75, MCA 2 = 0.15, MCA 3 = 0.33, MCA 4 = 0.23, and on chronograms, MCA 1 = 0.65, MCA 2 = 0.16, MCA 3 = 0.36, MCA 4 = 0.22.

### Morphological evolutionary rate

**Figure 6:**
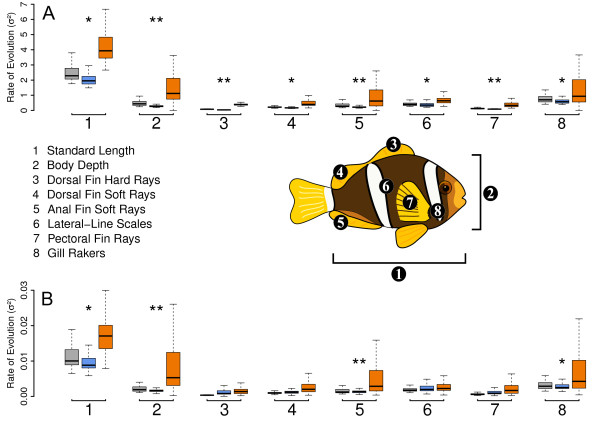
***Rate of morphological evolution. *** Evolution of the morphological traits measured on the sample of 100 phylograms **(A)** and chronograms **(B)**. Rates of clownfishes are in orange, damselfishes in blue and the one-rate model is shown in white. The P-values of model comparison by likelihood ratio test is signified by asterisk (* = P-values <0.05, ** = P-values <0.01). The schematic position of each morphological trait is shown on the clownfish drawing.

We measured, for each morphological trait, the rate parameter of a BM model of character evolution to assess if the appearance of mutualism was linked to an increased rate of morphological evolution in the clownfishes. We estimated, using AICc, if a model where clownfishes and damselfishes have distinct rates explains the observed data better than did a model of common rate between the two groups. We found that, on phylograms, all traits studied had a larger rate of evolution in the clownfishes than in the damselfishes (Figure 6). The pattern was more variable when measured on chronograms. In this case, only the standard length, body ratio, soft anal fin-rays and gill rakers had a faster evolutionary rate in the clownfishes. To verify that the elevated rates found in the clownfishes were not due to the relatively short branches of the clownfish clade we simulated a continuous trait under a BM model with a single rate on our phylogenies. The rates we recovered were not different between clownfishes and damselfishes.

## Discussion

**Figure 7:**
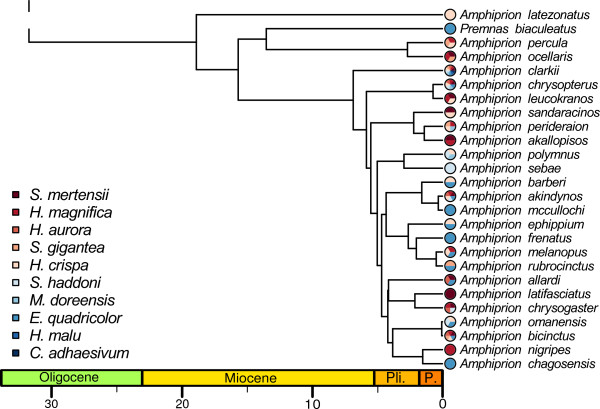
***Chronogram of the clownfishes radiation. *** Branch lengths are given in MY. The interacting sea anemone species are shown for each clownfish species. Sea anemone names abbreviations as in Figure 5.

We found that clownfishes exhibit patterns that are likely diagnostic of an ecological adaptive radiation via ecological speciation [4]. Following the acquisition of specific ability to interact and live with sea anemones, clownfishes diversified into multiple ecological niches linked with both host (Figure 7) and habitat use. Morphological evolution accelerated and distinct clownfish species developed convergent phenotypes correlated to the host-associated ecological niches.

### Pomacentridae phylogeny and diversification

Our inferred phylogeny was congruent with previous work [17,41] and showed with high posterior probabilities that clownfish are monophyletic within the Pomacentridae family (Figure 2). We used the BiSSE method [53] to test if the appearance of mutualism acted as a key innovation and if it is associated with an increase in speciation rate. The BiSSE method is a powerful way to detect shifts in diversification rate linked with a binary trait, but the change of state of the binary trait does not usually correspond to a single monophyletic group as in our case. Nevertheless, we chose the BiSSE method as it takes into account the uncertainty in dating the appearance of mutualism on the phylogeny. Indeed, mutualism with sea anemones likely appeared in the common ancestor of the clownfish, but the stem branch of the group is ~13MY long (Figure 2). It is therefore important, when estimating speciation and extinction rates, to take into account the uncertainty in the time estimates that correspond to the appearance of this behaviour. We also constrained the rate of mutualism loss to be null in the BiSSE model. This takes into account the fact that all clownfish are nowadays mutualistic, but it may slightly bias our analysis as it is not impossible that a clownfish species will eventually revert to a non-mutualistic state. However, we do not think that this parameter will influence significantly our results. Other methods exist to infer speciation rates on phylogenies that do not need prior hypotheses on the location of the shift in rate [64]. In a recent paper [41], the likely nodes of diversification rate shifts were inferred for four coral reef fish families using relative cladogenesis test [65] and MEDUSA [64]. The study included the Pomacentridae family and they consistently found across methods that the clownfishes experienced a significant rate increase. The rate shift was either placed at the origin of the clownfish or at the base of the *Amphiprion* crown group. The fact that a method which does not need *a priori* information on the location of the diversification rate shift recovered similar result as in our study confirms the strength of the diversification rate shift that occurred in the clownfish clade.

It should be noted that the extinction rate also increases in the clownfishes (Figure 3). A possible explanation is that during the diversification process, some clownfish lineages did not leave any descendants as they were ecologically replaced by other more competitive clownfish species. Such events could have occurred during the long branch that is basal to the *Amphiprion* crown group. Also, it has been suggested that elevated extinction rates in reef associated fishes could be linked with a potential refuge effect of the coral reefs in the aftermath of prolonged extinction events [41]. Yet, to be able to recover extinction rate with high confidence and test such hypotheses, one would need clownfishes fossils [66], which are not available. However, when compared to the damselfishes, the diversification rate of the clownfishes was still higher (Figure 3), showing that the extinction rate was not sufficient to slow down diversification [41].

### Effect of mutualism and host-associated niches on clownfishes evolution

We measured the phylogenetic signal of each morphological trait on the samples of chronograms and phylograms for the Pomacentridae (Tables 2 and 3). All traits showed a signal close to one (the expected outcome of BM evolution). However, when assessed only on the clownfish clade, phylogenetic signal dropped and only pectoral rays count had a K significantly larger than 0. Furthermore, the phylogenetic signal of the host usage MCA axes were never significantly different from 0. While interpreting an evolutionary process directly from this result can be problematic [67], a low phylogenetic signal can be found in lineages that show convergent adaptive evolution [68], which is likely the case in the clownfishes.

We hypothesised that, following the appearance of mutualism, clownfishes radiated in the niches associated with the sea anemones. We described the most important axes of variation in mutualistic interactions with an MCA. The first axis depicted the generalist-specialist host usage gradient, but all three other axes showed gradients linked with the habitat preferences of the sea anemones. Indeed, clownfishes that interact with sea anemones species living in similar reef micro-habitats (i.e. substrate type, depth) cluster together in the analysis. This suggests that clownfish species are first distributed along a generalist to specialist axis, and then, specialist clownfishes interact only with sea anemone species living in a particular habitat type. This has been shown in a previous empirical study [18], where clownfish species coexisting in a reef were distributed in different habitats. Ecological sorting of clownfish species along the different ecological gradients linked with their hosts is what is expected if resource competition, which is the main driver of adaptive radiation, acted on the evolutionary process [4,69].

We tested if the observed resource partitioning in different ecological niches, likely due to past competition between ecologically similar species, resulted in morphological adaptation to resource use (i.e. host and habitat use in clownfishes). We sequentially fitted each MCA axis to a set of morphological traits taking into account the phylogenetic relationships between species. We found that an important part of the variation in the MCA axes could be explained by the morphological traits of our dataset for the first and third axes (Table 4). Increasing number of hosts (represented by increasing values of the MCA 1 axis) was correlated with a bigger size, more hard dorsal and soft anal rays, and more gill rakers, while size was positively correlated with deeper habitats (represented by decreasing values of the MCA 3 axis). In clownfishes, the phenotype-environment correlation relates to both host usage (generalists/specialist gradient) and habitat (substrate, depth). This contrasts with many examples of adaptive radiation, where the resource axis has usually a single dimension representing habitat. Therefore, mutualism can be seen as a a key innovation that offered untapped habitat for colonisation, but also allowed diversification to happen on the host usage resource axes.

The morphological traits studied are primarily used for taxonomic purposes but they can still give important functional information for the evolution of the clownfishes. Size and fin traits are related to the locomotion ability in various types of water velocities, while gill rakers can be used as a proxy for the trophic level. The picture given by our analyses is that generalist clownfishes (e.g. *A. clarkii*) will likely eat more planktonic food (and thus have more gill rakers) and be better swimmers than specialists, which never leave the close vicinity of their sea anemone host. Clownfishes that interact with sea anemones occurring at deeper depth have also a bigger size, allowing for better locomotion in areas where the water velocity is likely to be higher compared to more shallow and sheltered zones [70]. While our results show correlations between phenotype and environment, we do not test for trait utility. This would require a strict experimental setting that was out of scope for this paper. More studies are definitely needed to better describe the adaptive advantage that those traits may provide in the ecological context of the mutualistic interaction.

Following an ecological opportunity, the rate of morphological evolution is hypothesised to be elevated in the traits that are functionally related to the ecological niches filled during the radiation process [4,71,72]. We tested this hypothesis on the eight morphological traits studied and found that, on phylograms, they all evolved at higher rates in clownfishes than in damselfishes (Figure 6). The picture is similar when rates are measured on chronograms, although only four characters are evolving significantly faster in the clownfishes than in the damselfishes in this case. Following the comparison of phylogenetic signal that was made between phylograms and chronograms, phylograms are assumed to give, in this case, more accurate results [51]. The fact that all traits evolve at a higher rate is congruent with the pGLS results, which shows all but two traits (lateral-line scales and body ratio) being significantly linked with host and habitat usage. It is probable that lateral-line scales and body ratio evolutionary rates were accelerated in clownfishes because they are indirectly correlated to the ecological niche through another trait. Thus they would not be themselves correlated to host-usage/habitat but still show accelerated rates of evolution. A clear followup to this broad description of clownfishes morphologies would be to extend our analysis and use a morphometric approach (e.g. [36]) to be able to give an accurate description of the different clownfishes ecotypes. Further studies will then be needed to test, in an experimental framework, trait utility, which is one of the diagnostic criteria of adaptive radiation [4]. The chemical biology of the interaction between clownfishes and sea anemones is also far from being solved [22]. It is thus possible that unknown characteristics associated for example, with the clownfishes mucus could be linked with the variation in interaction between clownfishes and specific sea anemones.

We did not take into account the distribution of the species in this study. Geographical isolation, coupled with ecological differentiation, could also be at the origin of the evolutionary pattern found here. While several clownfish species are local endemics that likely originated through vicariance events rather than ecological speciation, the majority of the species (17 out of 30) have overlapping distributions centred on the Indo-Malay archipelago. If reproductive isolation was solely due to geography, the latter species would have likely disappeared through hybridisation, which easily happens in captivity [73]. Moreover, clownfishes that have similar MCA values usually do not overlap in geographical distribution (e.g. *A. latifasciatus*, *A. nigripes* and *A. sandaracinos* in Figure 4), and sister species always differ in host usage as can be seen on Figure 7. Such a pattern could indicate that, in a given biogeographic region, only one species per ecological niche can subsist, but also that ecologically similar species evolved independently in geographically separated areas. This outlines the need for a thorough biogeographic analysis that would help clarify the effect of geography on the evolution of the clownfish.

## Conclusion

Our study shows that clownfishes likely experienced an adaptive radiation through ecological speciation. The obligate mutualism with sea anemones is thought to be the key innovation that allowed clownfishes to radiate rapidly in untapped ecological niches. As expected under the ecological theory of adaptive radiation [4], it increased diversification as well as rates of morphological evolution. Clownfishes experienced rapid and convergent morphological changes that were correlated with the different ecological niches offered by the host anemones. In marine environments, barriers to dispersal are uncommon, which makes ecological speciation less likely than in more isolated landscapes [25]. However clownfishes show a very short dispersal period compared to other damselfishes [74]. In conjunction with a high retention of larva to natal reef [27] and population specific calls [30], restricted dispersal likely reinforced reproductive isolation between clownfish species allowing for adaptive radiation.

## Competing interests

The authors declare that they have no competing interests.

## Authors’ contributions

GL and NS conceived the study. GL, CS and RW performed the analyses and drafted the manuscript. NS supervised GL, coordinated the project and helped to draft the manuscript. PB and NZ supervised RW, participated in the coordination and helped to draft the manuscript. All authors read and approved the final manuscript.

## Supplementary Material

Additional file 1 References of morphological data used in this study. Click here for file

Additional file 2 GenBank accession number of the sequences used in this study. Click here for file

Additional file 3 Substitution model choice. Click here for file

Additional file 4 Phylogeny of the damselfish with branch lengths given in expected number of substitutions per site. Click here for file

Additional file 5 Factorial map of the MCA analysis with eigenvectors for the axes 1 and 2. Click here for file

Additional file 6 Factorial map of the MCA analysis with eigenvectors for the axes 3 and 4. Click here for file
