# Supplementary material for: Mutualism with sea anemones triggered the adaptive radiation of clownfishes
Source: BMC Evol Biol. 2012 Nov 2;12:212. doi: 10.1186/1471-2148-12-212 (PMC3532366; doi:10.1186/1471-2148-12-212)
Supplement: Additional file 1 — References of morphological data used in this study. [file 1471-2148-12-212-S1.pdf]

References of morphological data used in this study

Abudefduf abdominalis	(Allen, 1991)
Abudefduf bengalensis	(Allen, 1991)
Abudefduf concolor	(Allen & Robertson, 1994)
Abudefduf declivifrons	(Allen, 1991)
Abudefduf hoefleri	(Allen, 1991)
Abudefduf lorenzi	(Allen, 1991)
Abudefduf luridus	(Allen, 1991)
Abudefduf margariteus	(Allen, 1991)
Abudefduf notatus	(Allen, 1991)
Abudefduf saxatilis	(Allen, 1991)
Abudefduf septemfasciatus	(Allen, 1991)
Abudefduf sexfasciatus	(Allen, 1991)
Abudefduf sordidus	(Allen, 1991)
Abudefduf sparoides	(Allen, 1991)
Abudefduf taurus	(Allen, 1991)
Abudefduf troschelii	(Allen, 1991)
Abudefduf vaigiensis	(Allen, 1991)
Abudefduf whitleyi	(Allen, 1991)
Acanthochromis polyacanthus	(Allen, 1991)
Altrichthys azurelineatus	(Allen, 1999)
Altrichthys curatus	(Allen, 1999)
Amblyglyphidodon aureus	(Allen, 1991)
Amblyglyphidodon curacao	(Allen, 1991)
Amblyglyphidodon leucogaster	(Allen, 1991)
Amblypomacentrus clarus	(Allen & Adrim 2000)
Amphiprion akallopisos	(Allen, 1991)
Amphiprion akindynos	(Allen, 1991)
Amphiprion allardi	(Allen, 1991)
Amphiprion barberi	(Allen et al,2008)
Amphiprion bicinctus	(Allen, 1991)
Amphiprion chagosensis	(Allen, 1991)
Amphiprion chrysogaster	(Allen, 1991)
Amphiprion chrysopterus	(Allen, 1991)
Amphiprion clarkii	(Allen, 1991)
Amphiprion ephippium	(Allen, 1991)
Amphiprion frenatus	(Allen, 1991)
Amphiprion latezonatus	(Allen, 1991)
Amphiprion latifasciatus	(Allen, 1991)
Amphiprion leucokranos	(Allen, 1991)
Amphiprion mccullochi	(Allen, 1991)
Amphiprion melanopus	(Allen, 1991)
Amphiprion nigripes	(Allen, 1991)
Amphiprion ocellaris	(Allen, 1991)
Amphiprion omanensis	(Allen, 1991)
Amphiprion percula	(Allen, 1991)
Amphiprion perideraion	(Allen, 1991)
Amphiprion polymnus	(Allen, 1991)

Amphiprion rubrocinctus	(Allen, 1991)
Amphiprion sandaracinos	(Allen, 1991)
Amphiprion sebae	(Allen, 1991)
Azurina hirundo	(Allen, 1991)
Cheiloprion labiatus	(Allen, 1991)
Chromis abyssus	(Pyle, Earle, & Greene, 2008)
Chromis acares	(Allen, 1991)
Chromis agilis	(Allen, 1991)
Chromis alpha	(Allen, 1991)
Chromis alta	(Allen, 1991)
Chromis amboinensis	(Allen, 1991)
Chromis analis	(Allen, 1991)
Chromis atrilobata	(Allen, 1991)
Chromis atripectoralis	(Allen, 1991)
Chromis atripes	(Allen, 1991)
Chromis brevirostris	(Pyle, Earle, & Greene, 2008)
Chromis cadenati	(Allen, 1991)
Chromis caerulea	(Allen, 1991)
Chromis caudalis	(Allen, 1991)
Chromis chromis	(Allen, 1991)
Chromis chrysura	(Allen, 1991)
Chromis circumaurea	(Pyle, Earle, & Greene, 2008)
Chromis cyanea	(Allen, 1991)
Chromis dasygenys	(Allen, 1991)
Chromis degruyi	(Pyle, Earle, & Greene, 2008)
Chromis flavomaculata	(Allen, 1991)
Chromis fumea	(Allen, 1991)
Chromis insolata	(Allen, 1991)
Chromis iomelas	(Allen, 1991)
Chromis limbata	(Allen, 1991)
Chromis margaritifer	(Allen, 1991)
Chromis multilineata	(Allen, 1991)
Chromis nitida	(Allen, 1991)
Chromis notata	(Allen, 1991)
Chromis opercularis	(Allen, 1991)
Chromis ovatiformis	(Allen, 1991)
Chromis punctipinnis	(Allen, 1991)
Chromis retrofasciata	(Allen, 1991)
Chromis ternatensis	(Allen, 1991)
Chromis vanderbilti	(Allen, 1991)
Chromis viridis	(Allen, 1991)
Chromis weberi	(Allen, 1991)
Chromis woodsi	(Allen, 1991)
Chromis xanthochira	(Allen, 1991)
Chromis xanthopterygia	(Allen, 1991)
Chromis xanthura	(Allen, 1991)
Chrysiptera annulata	(Allen, 1991)
Chrysiptera brownriggii	(Allen, 1991)
Chrysiptera caeruleolineata	(Allen, 1991)

Chrysiptera cyanea	(Allen, 1991)
Chrysiptera galba	(Allen, 1991)
Chrysiptera glauca	(Allen, 1991)
Chrysiptera hemicyanea	(Allen, 1991)
Chrysiptera kuiteri	(Allen, 1995)
Chrysiptera leucopoma	(Allen, 1991)
Chrysiptera oxycephala	(Allen, 1991)
Chrysiptera parasema	(Allen, 1991)
Chrysiptera rex	(Allen, 1991)
Chrysiptera rollandi	(Allen, 1991)
Chrysiptera springeri	(Allen, 1991)
Chrysiptera starcki	(Allen, 1991)
Chrysiptera talboti	(Allen, 1991)
Chrysiptera taupou	(Allen, 1991)
Chrysiptera unimaculata	(Allen, 1991)
Dascyllus albisella	(Allen, 1991)
Dascyllus aruanus	(Allen, 1991)
Dascyllus carneus	(Allen, 1991)
Dascyllus flavicaudus	(Allen, 1991)
Dascyllus marginatus	(Allen, 1991)
Dascyllus melanurus	(Allen, 1991)
Dascyllus reticulatus	(Allen, 1991)
Dascyllus strasburgi	(Allen, 1991)
Dascyllus trimaculatus	(Allen, 1991)
Dischistodus chrysopoecilus	(Allen, 1991)
Dischistodus melanotus	(Allen, 1991)
Dischistodus perspicillatus	(Allen, 1991)
Dischistodus prosopotaenia	(Allen, 1991)
Dischistodus pseudochrysopoecilus	(Allen, 1991)
Hemiglyphidodon plagiometopon	(Allen, 1991)
Hypsypops rubicundus	(Allen, 1991)
Lepidozygus tapeinosoma	(Allen, 1991)
Mecaenichthys immaculatus	(Allen, 1991)
Microspathodon chrysurus	(Allen, 1991)
Microspathodon dorsalis	(Allen, 1991)
Neoglyphidodon melas	(Allen, 1991)
Neoglyphidodon nigroris	(Allen, 1991)
Neoglyphidodon oxyodon	(Allen, 1991)
Neoglyphidodon polyacanthus	(Allen, 1991)
Neoglyphidodon thoracotaeniatus	(Allen, 1991)
Neopomacentrus azysron	(Allen, 1991)
Neopomacentrus cyanomos	(Allen, 1991)
Neopomacentrus filamentosus	(Allen, 1991)
Neopomacentrus miryae	(Allen, 1991)
Neopomacentrus nemurus	(Allen, 1991)
Neopomacentrus sindensis	(Allen, 1991)
Neopomacentrus taeniurus	(Allen, 1991)
Nexilosus latifrons	(Allen, 1991)
Parma microlepis	(Allen, 1991)

Parma oligolepis	(Allen, 1991)
Plectroglyphidodon dickii	(Allen, 1991)
Plectroglyphidodon lacrymatus	(Allen, 1991)
Plectroglyphidodon leucozonus	(Allen, 1991)
Pomacentrus adelus	(Allen, 1991)
Pomacentrus albicaudatus	(Allen, 1991)
Pomacentrus alexanderae	(Allen, 1991)
Pomacentrus alleni	(Allen, 1991)
Pomacentrus amboinensis	(Allen, 1991)
Pomacentrus auriventris	(Allen, 1991)
Pomacentrus australis	(Allen, 1991)
Pomacentrus bankanensis	(Allen, 1991)
Pomacentrus brachialis	(Allen, 1991)
Pomacentrus burroughi	(Allen, 1991)
Pomacentrus caeruleus	(Allen, 1991)
Pomacentrus chrysurus	(Allen, 1991)
Pomacentrus coelestis	(Allen, 1991)
Pomacentrus grammorhynchus	(Allen, 1991)
Pomacentrus lepidogenys	(Allen, 1991)
Pomacentrus leptus	(Allen, 1991)
Pomacentrus milleri	(Allen, 1991)
Pomacentrus moluccensis	(Allen, 1991)
Pomacentrus nagasakiensis	(Allen, 1991)
Pomacentrus nigromanus	(Allen, 1991)
Pomacentrus nigromarginatus	(Allen, 1991)
Pomacentrus pavo	(Allen, 1991)
Pomacentrus philippinus	(Allen, 1991)
Pomacentrus reidi	(Allen, 1991)
Pomacentrus smithi	(Allen, 1991)
Pomacentrus stigma	(Allen, 1991)
Pomacentrus trichourus	(Allen, 1991)
Pomacentrus trilineatus	(Allen, 1991)
Pomacentrus vaiuli	(Allen, 1991)
Pomachromis fuscidorsalis	(Allen, 1991)
Pomachromis richardsoni	(Allen, 1991)
Premnas biaculeatus	(Allen, 1991)
Pristotis obtusirostris	(Randall et al.,1997)
Similiparma hermani	(Allen, 1991)
Stegastes adustus	(Allen, 1991)
Stegastes altus	(Allen, 1991)
Stegastes apicalis	(Allen, 1991)
Stegastes diencaeus	(Allen, 1991)
Stegastes fasciolatus	(Allen, 1991)
Stegastes imbricatus	(Allen, 1991)
Stegastes leucostictus	(Allen, 1991)
Stegastes lividus	(Allen, 1991)
Stegastes nigricans	(Allen, 1991)
Stegastes obreptus	(Allen, 1991)
Stegastes partitus	(Allen, 1991)

Stegastes planifrons	(Allen, 1991)
Stegastes variabilis	(Allen, 1991)
Teixeirichthys jordani	(Allen, 1991)

Allen, G.R. 1975. Damselfishes of the south seas. T.F.H. Publications, Neptune city.

Allen, G.R. 1991. Damselfish of the world. Mergus Publishers, Melle, Germany.

Allen, G.R. 1999. Altrichthys, a New Genus of Damselfish (Pomacentridae) from Philippine Seas with Description of a New Species. Revue fr. Aquariol. 26: 23-28.

Allen, G.R., J. Drew, and L. Kaufman. 2008. Amphiprion barberi , a new species of anemonefish (Pomacentridae) from Fiji , Tonga , and Samoa. aqua, International Journal of Ichthyology 14: 105-114.

Allen, G.R., and M. Adrim. 2000. Amblypomacentrus clarus , a new species of damselfish (Pomacentridae) from the Banggai Islands , Indonesia. Records of the Western Australian Museum 20: 51-55.

Pyle, R.L., J.L. Earle, and B.D. Greene. 2008. Five new species of the damselfish genus Chromis (Perciformes: Labroidei:Pomacentridae) from deep coral reefs in the tropical western Pacific. Zootaxa 1671: 3-31.

Randall, J.E., G. R. Allen and R. C. Steene. Fishes of the Great Barrier Reef and Coral Sea, 1997. University of Hawaii Press, Honolulu.
