# Supplementary material for: Mutualism with sea anemones triggered the adaptive radiation of clownfishes
Source: BMC Evol Biol. 2012 Nov 2;12:212. doi: 10.1186/1471-2148-12-212 (PMC3532366; doi:10.1186/1471-2148-12-212)
Supplement: Additional file 2 — GenBank accession number of the sequences used in this study. [file 1471-2148-12-212-S2.pdf]

GenBank accession number of the sequences used in this study

	12S	16S	ATP	bmp-4	COI	cytb	ND3	rag-1	rag-2
Abudefduf abdominalis	NA	NA	AY208403	NA	NA	AY208546	NA	NA	NA
Abudefduf bengalensis	FJ616289	FJ616397	AY208404	FJ616836	NA	AY208547	FJ616505	FJ616617	FJ616729
Abudefduf concolor	NA	NA	AY208405	NA	NA	AY208548	NA	NA	NA
Abudefduf declivifrons	NA	NA	AY208406	NA	NA	AY208549	NA	NA	NA
Abudefduf hoefleri	FJ616290	FJ616398	NA	FJ616837	NA	NA	FJ616506	FJ616618	FJ616730
Abudefduf lorenzi	NA	NA	AY208407	NA	NA	AY208550	NA	NA	NA
Abudefduf luridus	FJ616291	FJ616399	NA	FJ616838	NA	EF439185	FJ616507	FJ616619	FJ616731
Abudefduf margariteus	NA	NA	AY208408	NA	NA	AY208551	NA	AY208618	NA
Abudefduf notatus	FJ616292	FJ616400	AY208409	FJ616839	HQ561510	AY208552	FJ616508	FJ616620	FJ616732
Abudefduf saxatilis	FJ616293	AY279673	AY208410	FJ616840	GU225083	AY208553	FJ616509	AY208624	AY279879
Abudefduf septemfasciatus	FJ616294	FJ616402	AY208411	FJ616841	EF607296	AY208554	FJ616510	AY208619	FJ616734
Abudefduf sexfasciatus	FJ616295	FJ616403	AY208412	FJ616842	NA	AY208555	FJ616511	AY208620	FJ616735
Abudefduf sordidus	FJ616296	FJ616404	AY208413	FJ616843	NA	AY208556	FJ616512	AY208621	FJ616736
Abudefduf sparoides	NA	NA	AY208414	NA	NA	AY208558	NA	NA	NA
Abudefduf taurus	FJ616297	FJ616405	AY208415	FJ616844	NA	AY208559	FJ616513	AY208622	FJ616737
Abudefduf troschelii	NA	NA	AY208416	NA	NA	AY208560	NA	AY208623	NA
Abudefduf vaigiensis	AB121234	NA	AY208418	NA	FJ237571	AY208561	NA	NA	NA
Abudefduf whitleyi	FJ616298	FJ616406	AY208419	FJ616845	NA	AY208562	FJ616514	FJ616626	FJ616738
Acanthochromis polyacanthus	FJ616299	FJ616407	AY208369	FJ616846	NA	AY208521	FJ616515	AY208625	FJ616739
Altrichthys azurelineatus	NA	HQ629880	NA	NA	NA	NA	NA	HQ629886	HQ629896
Altrichthys curatus	FJ616300	FJ616408	NA	NA	NA	NA	NA	HQ629891	HQ629900
Amblyglyphidodon aureus	FJ616301	EF419265	AY208420	FJ616848	NA	AY208563	FJ616516	AY208627	FJ616741
Amblyglyphidodon curacao	FJ616302	EF419264	AY208422	FJ616849	NA	AY208564	FJ616517	FJ616629	FJ616742
Amblyglyphidodon leucogaster	AY279568	EF419267	AY208425	FJ616850	JF952668	AY208565	FJ616518	AY208626	AY279877
Amblypomacentrus clarus	FJ616304	FJ616412	NA	FJ616851	NA	NA	FJ616519	FJ616631	FJ616744
Amphiprion akallopisos	NA	NA	AY208344	NA	FJ582728	AY208508	NA	NA	NA
Amphiprion akindynos	NA	DQ343905	AY208346	NA	NA	AY208509	NA	AY208628	NA
Amphiprion allardi	NA	NA	AY208348	NA	NA	AY208510	NA	AY208629	NA
Amphiprion barberi	NA	NA	NA	NA	NA	NA	NA	NA	EU256991
Amphiprion bicinctus	NA	DQ343906	NA	NA	NA	DQ343946	NA	NA	NA
Amphiprion chagosensis	NA	DQ343907	NA	NA	NA	DQ343947	NA	NA	NA
Amphiprion chrysogaster	NA	NA	AY208350	NA	FJ582743	AY208511	NA	NA	NA
Amphiprion chrysopterus	NA	DQ343908	AY208352	NA	FJ582755	AY208512	NA	NA	NA
Amphiprion clarkii	FJ616305	FJ616413	AY208354	FJ616852	FJ582757	AY208513	FJ616520	FJ616632	FJ616745
Amphiprion ephippium	NA	NA	NA	NA	NA	JQ314455	NA	NA	NA
Amphiprion frenatus	FJ616306	FJ616414	NA	FJ616853	FJ582759	AF097930	FJ616521	FJ616633	FJ616746
Amphiprion latezonatus	NA	DQ343912	NA	NA	NA	NA	NA	NA	NA
Amphiprion latifasciatus	NA	NA	NA	NA	FJ582760	NA	NA	NA	NA
Amphiprion leucokranos	NA	DQ343913	NA	NA	NA	DQ343953	NA	NA	NA
Amphiprion mccullochi	NA	DQ343914	NA	NA	NA	DQ343954	NA	NA	NA
Amphiprion melanopus	FJ616307	FJ616415	AY208356	FJ616854	FJ582773	AY208514	FJ616522	FJ616634	FJ616747
Amphiprion nigripes	NA	NA	AY208358	NA	FJ582781	AY208515	NA	NA	NA
Amphiprion ocellaris	FJ616308	FJ616416	NA	FJ616855	FJ582789	AY208516	FJ616523	AY208631	FJ616748
Amphiprion omanensis	NA	NA	AY208362	NA	NA	AY208517	NA	NA	NA
Amphiprion percula	AF285924	AF285946	NA	NA	NA	DQ343958	NA	NA	NA
Amphiprion perideraion	FJ616309	FJ616417	AY208364	FJ616856	FJ582797	AY208518	FJ616524	AY208630	FJ616749
Amphiprion polymnus	NA	AY666170	NA	NA	FJ582813	AF097928	NA	NA	NA
Amphiprion rubrocinctus	NA	DQ343920	NA	NA	NA	DQ343961	NA	NA	NA
Amphiprion sandaracinos	FJ616310	FJ616418	NA	FJ616857	FJ582815	AF097929	FJ616525	FJ616637	FJ616750

Amphiprion sebae	NA	HQ020377	AY208366	NA	FJ582825	AY208519	NA	NA	NA
Azurina hirundo	FJ616311	FJ616419	NA	FJ616858	GU440243	NA	FJ616526	FJ616638	FJ616751
Cheiloprion labiatus	FJ616312	FJ616420	NA	FJ616859	NA	NA	FJ616527	FJ616639	FJ616752
Chromis abyssus	NA	NA	NA	NA	EU358598	NA	NA	NA	NA
Chromis acares	NA	NA	NA	NA	JN107891	NA	NA	NA	NA
Chromis agilis	NA	NA	AY208370	NA	HQ561507	AY208522	NA	AY208641	NA
Chromis alpha	FJ616313	FJ616421	NA	FJ616860	NA	NA	FJ616528	FJ616640	FJ616753
Chromis alta	NA	AY958642	NA	NA	GU440280	NA	NA	NA	NA
Chromis amboinensis	FJ616314	FJ616422	AY208372	FJ616861	NA	AY208523	FJ616529	FJ616641	FJ616754
Chromis analis	FJ616315	FJ616423	NA	FJ616862	NA	NA	FJ616530	FJ616642	FJ616755
Chromis atrilobata	NA	EF489730	AY208374	NA	NA	AY208524	NA	AY208637	NA
Chromis atripectoralis	FJ616316	FJ616424	AY208376	FJ616863	FJ583145	AY208525	FJ616531	FJ616643	FJ616756
Chromis atripes	NA	NA	AY208378	NA	NA	AY208526	NA	AY208633	NA
Chromis brevirostris	NA	NA	NA	NA	EU358580	NA	NA	NA	NA
Chromis cadenati	NA	NA	NA	NA	GQ341589	NA	NA	NA	NA
Chromis caerulea	NA	NA	NA	NA	FJ583147	NA	NA	NA	NA
Chromis caudalis	NA	NA	NA	NA	NA	AY289557	NA	NA	NA
Chromis chromis	FJ616317	FJ616425	AY208380	FJ616864	NA	AY208527	FJ616532	AY208640	FJ616757
Chromis chrysura	NA	NA	NA	NA	NA	AY208528	NA	NA	NA
Chromis circumaurea	NA	NA	NA	NA	EU358586	NA	NA	NA	NA
Chromis cyanea	AF285925	AF285947	AY208384	NA	FJ583153	AY208529	NA	AY208639	NA
Chromis dasygenys	NA	NA	NA	NA	HQ945824	NA	NA	NA	NA
Chromis degruyi	NA	NA	NA	NA	EU358590	NA	NA	NA	NA
Chromis flavomaculata	NA	NA	AY208386	NA	NA	AY208530	NA	NA	NA
Chromis fumea	AF081224	AY365121	NA	NA	NA	EU267219	NA	NA	NA
Chromis insolata	NA	NA	NA	NA	FJ583157	NA	NA	NA	NA
Chromis iomelas	AF285926	AF285948	AY208388	NA	JN107893	AY208531	NA	NA	NA
Chromis limbata	NA	EF489738	NA	NA	NA	EF439193	NA	NA	NA
Chromis margaritifer	FJ616318	FJ616426	AY208390	FJ616865	FJ583161	AY208532	FJ616533	FJ616645	FJ616758
Chromis multilineata	FJ616319	FJ616427	AY208392	FJ616866	GU224766	AY208533	FJ616534	AY208636	FJ616759
Chromis nitida	NA	NA	AY208394	NA	NA	AY208534	NA	AY208638	NA
Chromis notata	NA	NA	NA	NA	JF952702	EU267220	NA	NA	NA
Chromis opercularis	FJ616320	FJ616428	NA	FJ616867	HQ561474	NA	FJ616535	FJ616647	FJ616760
Chromis ovatiformis	FJ616321	FJ616429	NA	FJ616868	NA	NA	FJ616536	FJ616648	FJ616761
Chromis punctipinnis	FJ616322	FJ616430	NA	FJ616869	GU440281	AY289559	FJ616537	FJ616649	FJ616762
Chromis retrofasciata	FJ616323	FJ616431	AY208396	FJ616870	NA	AY208535	FJ616538	AY208634	FJ616763
Chromis ternatensis	FJ616324	FJ616432	NA	FJ616871	NA	NA	FJ616539	FJ616651	FJ616764
Chromis vanderbilti	NA	NA	NA	NA	JN107905	NA	NA	NA	NA
Chromis viridis	FJ616325	FJ616433	AY208398	FJ616872	FJ583167	AY208536	FJ616540	AY208635	FJ616765
Chromis weberi	FJ616326	FJ616434	AY208400	FJ616873	NA	AY208537	FJ616541	AY208642	FJ616766
Chromis woodsi	NA	NA	NA	NA	HM421816	NA	NA	NA	NA
Chromis xanthochira	FJ616327	FJ616435	NA	FJ616874	NA	AY289561	FJ616542	FJ616654	FJ616767
Chromis xanthopterygia	NA	NA	AY208402	NA	NA	AY208538	NA	NA	NA
Chromis xanthura	FJ616328	FJ616436	NA	FJ616875	NA	NA	FJ616543	FJ616655	FJ616768
Chrysiptera annulata	NA	NA	AY208426	NA	NA	AY208566	NA	AY208645	NA
Chrysiptera brownriggii	FJ616329	FJ616437	NA	FJ616876	NA	NA	FJ616544	FJ616656	FJ616769
Chrysiptera caeruleolineata	NA	NA	NA	NA	FJ459575	NA	NA	NA	NA
Chrysiptera cyanea	FJ616330	FJ616438	AY208428	FJ616877	FJ583181	AB018992	FJ616545	AY208643	FJ616770
Chrysiptera galba	NA	NA	AY208430	NA	FJ583183	AY208568	NA	NA	NA
Chrysiptera glauca	NA	NA	AY208432	NA	NA	AY208569	NA	AY208647	NA
Chrysiptera hemicyanea	NA	NA	AY208434	NA	NA	AY208570	NA	AY208644	NA
Chrysiptera kuiteri	FJ616336	FJ616444	NA	FJ616883	NA	NA	FJ616551	FJ616663	FJ616776
Chrysiptera leucopoma	AF081226	NA	AY208436	NA	NA	AY208571	NA	AY208648	NA

Chrysiptera oxycephala	FJ616331	FJ616439	NA	FJ616878	NA	NA	FJ616546	FJ616658	FJ616771
Chrysiptera parasema	NA	NA	NA	NA	FJ583192	NA	NA	NA	NA
Chrysiptera rex	FJ616332	FJ616440	AY208438	FJ616879	FJ583195	AY208572	FJ616547	FJ616659	FJ616772
Chrysiptera rollandi	FJ616333	EF419269	AY208440	FJ616880	NA	AY208573	FJ616548	AY208646	FJ616773
Chrysiptera springeri	FJ616334	FJ616442	NA	FJ616881	NA	NA	FJ616549	FJ616661	FJ616774
Chrysiptera starcki	NA	NA	NA	NA	FJ583201	NA	NA	NA	NA
Chrysiptera talboti	FJ616335	FJ616443	AY208442	FJ616882	FJ583204	AY208574	FJ616550	FJ616662	FJ616775
Chrysiptera taupou	NA	NA	AY208444	NA	FJ583212	AY208575	NA	NA	NA
Chrysiptera unimaculata	FJ616337	FJ616445	NA	FJ616884	NA	NA	FJ616552	FJ616664	FJ616777
Dascyllus albisella	NA	AF119405	AF489746	NA	NA	AF119396	NA	NA	NA
Dascyllus aruanus	FJ616338	FJ616446	AF489764	FJ616885	FJ583323	AY208539	FJ616553	AY208649	FJ616778
Dascyllus carneus	NA	AF119404	AF489774	NA	NA	AY208540	NA	AY208652	NA
Dascyllus flavicaudus	NA	AF119407	AF489780	NA	FJ583325	AY208541	NA	AY208650	NA
Dascyllus marginatus	NA	AF119403	AF489784	NA	NA	AY208542	NA	AY208651	NA
Dascyllus melanurus	FJ616339	FJ616447	AF489788	FJ616886	FJ583327	AY208543	FJ616554	AY208653	FJ616779
Dascyllus reticulatus	FJ616340	FJ616448	AF489804	FJ616887	FJ583332	AY208544	FJ616555	AY208654	FJ616780
Dascyllus strasburgi	NA	NA	AF489808	NA	NA	NA	NA	NA	NA
Dascyllus trimaculatus	AY751750	FJ616449	AF489822	FJ616888	FJ583337	NA	FJ616556	AY208655	AY279878
Dischistodus chrysopoecilus	AY279567	AY279670	NA	FJ616889	NA	NA	FJ616557	FJ616669	AY279876
Dischistodus melanotus	FJ616343	FJ616451	AY208445	FJ616890	NA	AY208576	FJ616558	AY208656	FJ616783
Dischistodus perspicillatus	FJ616344	FJ616452	NA	FJ616891	NA	NA	FJ616559	FJ616671	FJ616784
Dischistodus prosopotaenia	FJ616345	FJ616453	NA	FJ616892	FJ583365	NA	FJ616560	FJ616672	FJ616785
Dischistodus pseudochrysopoecilus	FJ616346	FJ616454	NA	FJ616893	FJ583369	NA	FJ616561	FJ616673	FJ616786
Hemiglyphidodon plagiometopon	FJ616347	FJ616455	AY208446	FJ616894	NA	AY208577	FJ616562	FJ616674	FJ616787
Hypsypops rubicundus	FJ616348	FJ616456	NA	FJ616895	JN600313	NA	FJ616563	FJ616675	FJ616788
Lepidozygus tapeinosoma	FJ616349	FJ616457	NA	FJ616896	NA	NA	FJ616564	FJ616676	FJ616789
Mecaenichthys immaculatus	FJ616350	FJ616458	NA	FJ616897	NA	NA	FJ616565	FJ616677	FJ616790
Microspathodon chrysurus	FJ616351	FJ616459	AY208448	FJ616898	FJ583660	AY208578	FJ616566	AY208657	FJ616791
Microspathodon dorsalis	FJ616352	FJ616460	NA	FJ616899	NA	NA	FJ616567	FJ616679	FJ616792
Neoglyphidodon melas	FJ616353	FJ616461	AY208450	FJ616900	FJ583726	AY208579	FJ616568	FJ616680	FJ616793
Neoglyphidodon nigroris	FJ616354	FJ616462	AY208452	FJ616901	FJ583729	AY208580	FJ616569	AY208658	FJ616794
Neoglyphidodon oxyodon	FJ616355	FJ616463	AY208453	FJ616902	FJ583736	AY208581	FJ616570	AY208659	FJ616795
Neoglyphidodon polyacanthus	AF285931	AF285953	AY208454	NA	NA	AY208582	NA	NA	NA
Neoglyphidodon thoracotaeniatus	FJ616356	FJ616464	NA	FJ616903	NA	NA	FJ616571	FJ616683	FJ616796
Neopomacentrus azysron	FJ616357	FJ616465	NA	FJ616904	NA	NA	FJ616572	FJ616684	FJ616797
Neopomacentrus cyanomos	AY098626	AY098631	AY208455	NA	NA	AY208583	NA	NA	NA
Neopomacentrus filamentosus	NA	NA	AY208456	NA	NA	AY208584	NA	AY208661	NA
Neopomacentrus miryae	NA	NA	AY208458	NA	NA	AY208585	NA	NA	NA
Neopomacentrus nemurus	NA	NA	AY208460	NA	NA	AY208586	NA	NA	NA
Neopomacentrus sindensis	NA	NA	AY208462	NA	NA	AY208587	NA	AY208660	NA
Neopomacentrus taeniurus	FJ616358	FJ616466	NA	FJ616905	NA	NA	FJ616573	FJ616685	FJ616798
Nexilosus latifrons	FJ616359	FJ616467	NA	NA	NA	NA	NA	NA	NA
Parma microlepis	FJ616360	FJ616468	NA	FJ616907	NA	NA	FJ616574	FJ616686	FJ616799
Parma oligolepis	AF285932	AF285954	AY208464	NA	NA	AY208588	NA	AY208662	NA
Plectroglyphidodon dickii	FJ616361	FJ616469	AY208466	FJ616908	NA	AY208589	FJ616575	AY208663	FJ616800
Plectroglyphidodon lacrymatus	FJ616362	FJ616470	NA	FJ616909	NA	NA	FJ616576	FJ616688	FJ616801
Plectroglyphidodon leucozonus	FJ616363	FJ616471	AY208468	FJ616910	HQ945825	AY208590	FJ616577	FJ616689	FJ616802
Pomacentrus adelus	NA	NA	AY208470	NA	NA	AY208591	NA	AY208664	NA
Pomacentrus albicaudatus	FJ616364	FJ616472	NA	FJ616911	NA	NA	FJ616578	FJ616690	FJ616803
Pomacentrus alexanderae	FJ616365	FJ616473	NA	FJ616912	NA	NA	FJ616579	FJ616691	FJ616804
Pomacentrus alleni	NA	NA	NA	NA	FJ583889	NA	NA	NA	NA
Pomacentrus amboinensis	FJ616366	FJ616474	NA	FJ616913	NA	NA	FJ616580	FJ616692	FJ616805
Pomacentrus australis	NA	NA	AY208472	NA	NA	AY208592	NA	AY208668	NA

Pomacentrus bankanensis	FJ616367	AY365122	AY208473	FJ616914	NA	AY208593	FJ616581	AY208665	FJ616806
Pomacentrus brachialis	FJ616368	FJ616476	AY208474	FJ616915	NA	AY208594	FJ616582	FJ616694	FJ616807
Pomacentrus burroughi	FJ616369	FJ616477	NA	FJ616916	NA	NA	FJ616583	FJ616695	FJ616808
Pomacentrus caeruleus	NA	NA	NA	NA	FJ583896	NA	NA	NA	NA
Pomacentrus chrysurus	FJ616370	FJ616478	AY208476	FJ616917	NA	AY208595	FJ616584	FJ616696	FJ616809
Pomacentrus coelestis	FJ616371	AY365123	AY208478	FJ616918	FJ583898	AY208596	FJ616585	FJ616697	FJ616810
Pomacentrus grammorhynchus	FJ616372	FJ616480	AY208480	FJ616919	NA	AY208597	FJ616586	FJ616698	FJ616811
Pomacentrus lepidogenys	FJ616373	FJ616481	AY208482	FJ616920	NA	AY208598	FJ616587	FJ616699	FJ616812
Pomacentrus leptus	NA	NA	AY208484	NA	NA	AY208599	NA	NA	NA
Pomacentrus milleri	NA	NA	AY208485	NA	NA	AY208600	NA	NA	NA
Pomacentrus moluccensis	FJ616374	EF419268	AY208486	FJ616921	FJ583906	AY208601	FJ616588	AY208669	EU256721
Pomacentrus nagasakiensis	NA	NA	AY208488	NA	FJ583909	AY208602	NA	NA	NA
Pomacentrus nigromanus	FJ616375	FJ616483	AY208490	FJ616922	NA	AY208603	FJ616589	AY208671	FJ616814
Pomacentrus nigromarginatus	FJ616376	FJ616484	NA	FJ616923	NA	NA	FJ616590	FJ616702	FJ616815
Pomacentrus pavo	EF095577	EF095605	AY208492	NA	NA	AY208604	NA	AY208666	NA
Pomacentrus philippinus	FJ616377	FJ616485	AY208494	FJ616924	NA	AY208605	FJ616591	AY208667	FJ616816
Pomacentrus reidi	NA	NA	AY208496	NA	NA	AY208606	NA	AY208672	NA
Pomacentrus smithi	NA	NA	AY208498	NA	NA	AY208607	NA	AY208670	NA
Pomacentrus stigma	FJ616378	FJ616486	NA	FJ616925	NA	NA	FJ616592	FJ616704	FJ616817
Pomacentrus trichourus	NA	NA	NA	NA	HQ561512	NA	NA	NA	NA
Pomacentrus trilineatus	NA	NA	AY208500	NA	NA	AY208608	NA	NA	NA
Pomacentrus vaiuli	FJ616379	FJ616487	AY208502	FJ616926	NA	AY208609	FJ616593	FJ616705	FJ616818
Pomachromis fuscidorsalis	NA	NA	AY208503	NA	NA	AY208610	NA	NA	NA
Pomachromis richardsoni	FJ616380	FJ616488	NA	FJ616927	NA	NA	FJ616594	FJ616706	FJ616819
Premnas biaculeatus	FJ616381	FJ616489	AY208367	FJ616928	FJ583914	AY208520	FJ616595	AY208632	FJ616820
Pristotis obtusirostris	FJ616382	FJ616490	NA	FJ616929	NA	NA	FJ616596	FJ616708	FJ616821
Similiparma hermani	FJ616383	FJ616491	NA	FJ616930	NA	NA	FJ616597	FJ616709	FJ616822
Stegastes adustus	AF285937	AF285959	NA	NA	GU224603	NA	NA	NA	NA
Stegastes altus	AF081243	NA	NA	NA	NA	NA	NA	NA	NA
Stegastes apicalis	FJ616384	FJ616492	NA	FJ616931	NA	NA	FJ616598	FJ616710	FJ616823
Stegastes diencaeus	FJ616385	FJ616493	NA	FJ616932	GU225468	NA	FJ616599	FJ616711	FJ616824
Stegastes fasciolatus	FJ616386	FJ616494	NA	FJ616933	NA	NA	FJ616600	FJ616712	FJ616825
Stegastes imbricatus	FJ616387	FJ616495	NA	FJ616934	NA	NA	FJ616601	FJ616713	FJ616826
Stegastes leucostictus	FJ616388	FJ616496	NA	FJ616935	GU225476	NA	FJ616602	FJ616714	FJ616827
Stegastes lividus	AF081245	NA	NA	NA	NA	NA	NA	NA	NA
Stegastes nigricans	FJ616389	NA	NA	FJ616936	NA	NA	FJ616603	FJ616715	FJ616828
Stegastes obreptus	FJ616390	FJ616498	NA	FJ616937	NA	NA	FJ616604	FJ616716	FJ616829
Stegastes partitus	FJ616391	FJ616499	NA	FJ616938	GU225482	NA	FJ616605	FJ616717	FJ616830
Stegastes planifrons	NA	NA	AY208504	NA	GU224623	AY208611	NA	AY208673	NA
Stegastes variabilis	AF285938	AF285960	NA	NA	NA	NA	NA	NA	NA
Teixeirichthys jordani	FJ616392	FJ616500	NA	FJ616939	NA	NA	FJ616606	FJ616718	FJ616831
Aequidens rivulatus	NA	EF432886	NA	NA	EU888042	GU736965	NA	EU706379	GU736833
Tomocichla sieboldii	NA	DQ119179	NA	NA	DQ119208	AY843430	NA	EF362610	NA
Thorichthys meeki	AY279566	GU817230	NA	FJ616947	JN028435	HM193445	FJ616616	EF362590	AY279875
