# Supplementary figures and images for: Mutualism with sea anemones triggered the adaptive radiation of clownfishes
Source: BMC Evol Biol. 2012 Nov 2;12:212. doi: 10.1186/1471-2148-12-212 (PMC3532366; doi:10.1186/1471-2148-12-212)

12S

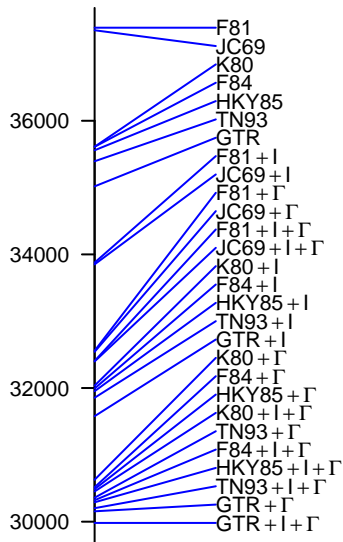

16S

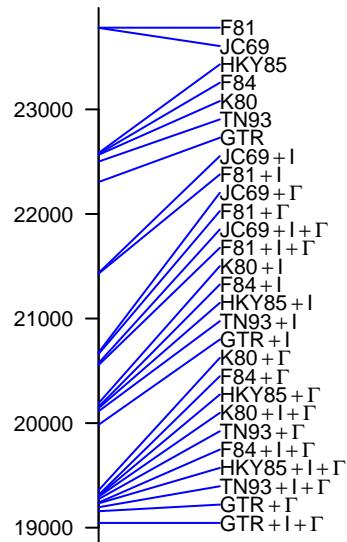

ATP

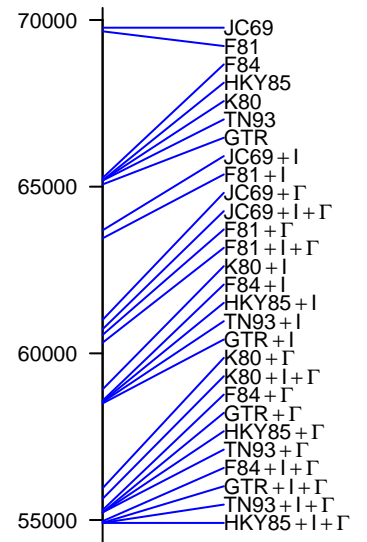

bmp-4

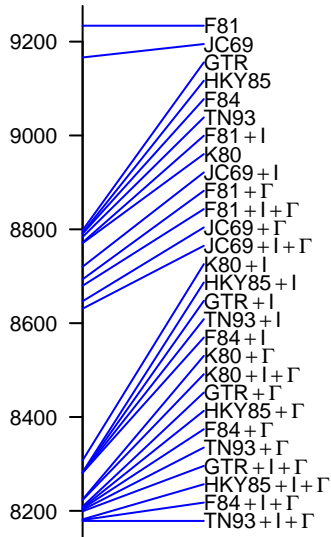

COI

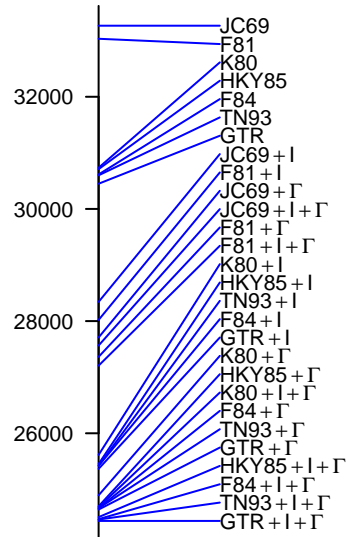

cytb

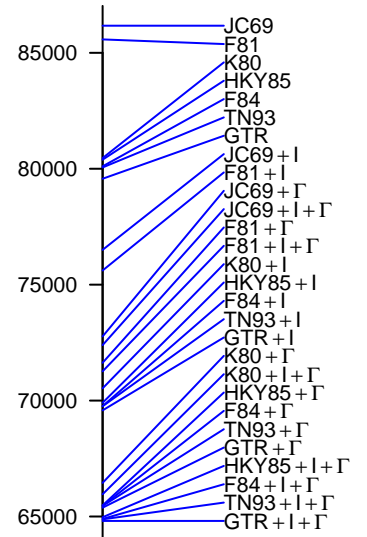

ND3

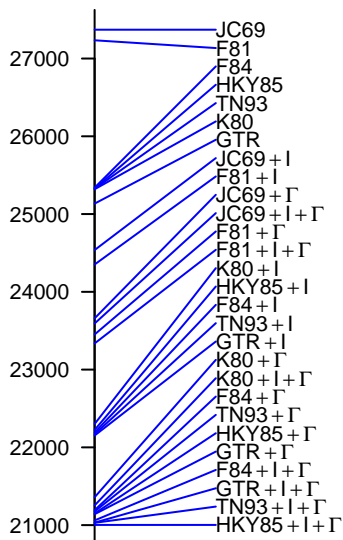

rag-1

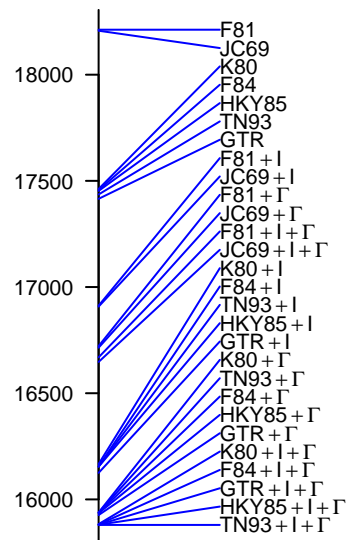

rag-2

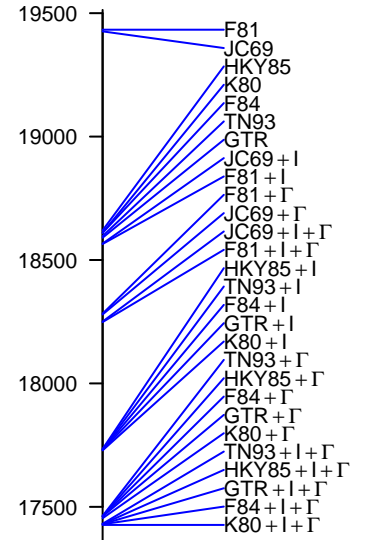

Supplement: Additional file 3 — Substitution model choice. [file 1471-2148-12-212-S3.pdf]

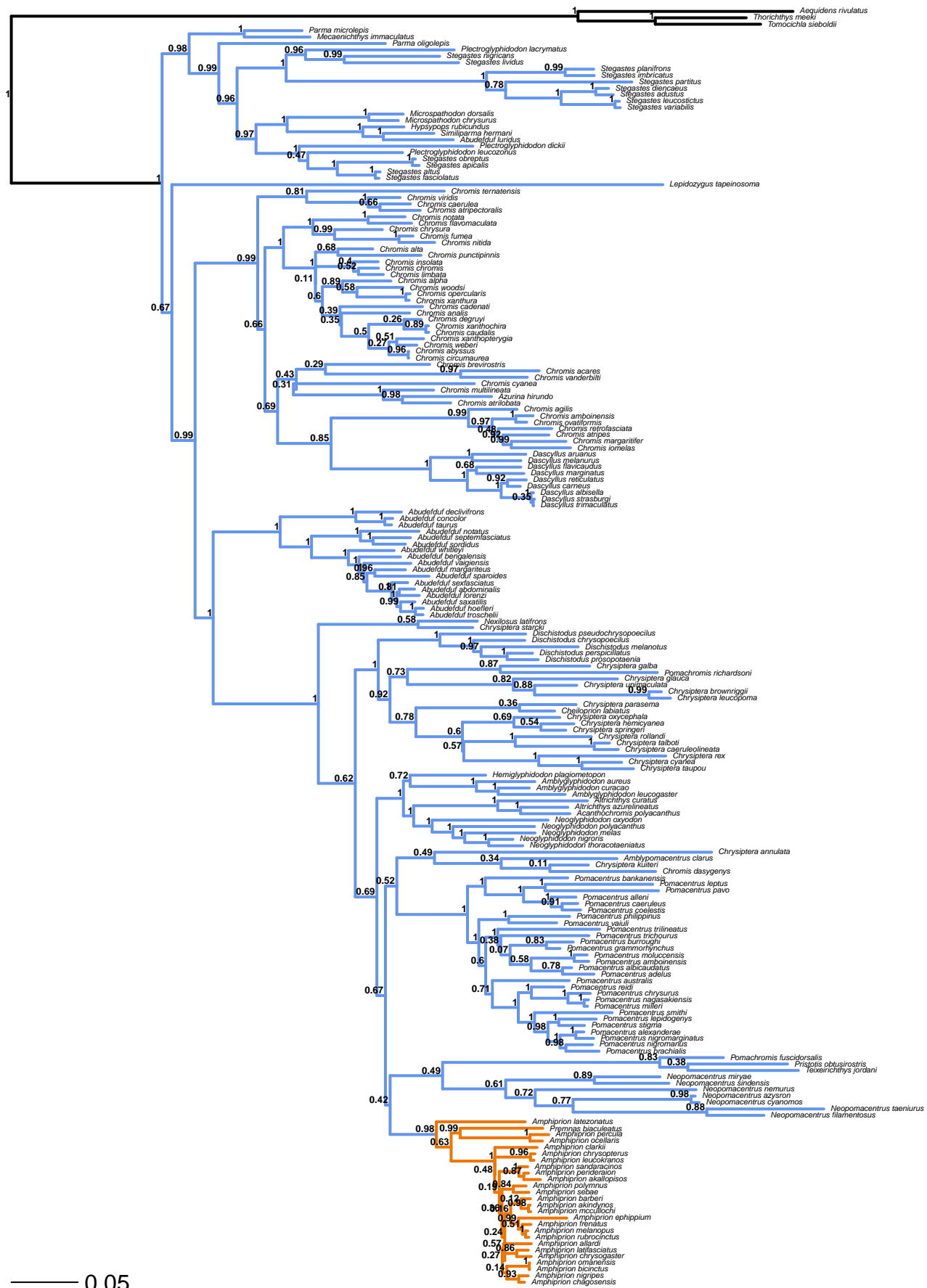

0.05

Supplement: Additional file 4 — Phylogeny of the damselfish with branch lengths given in expected number of substitutions per site. [file 1471-2148-12-212-S4.pdf]

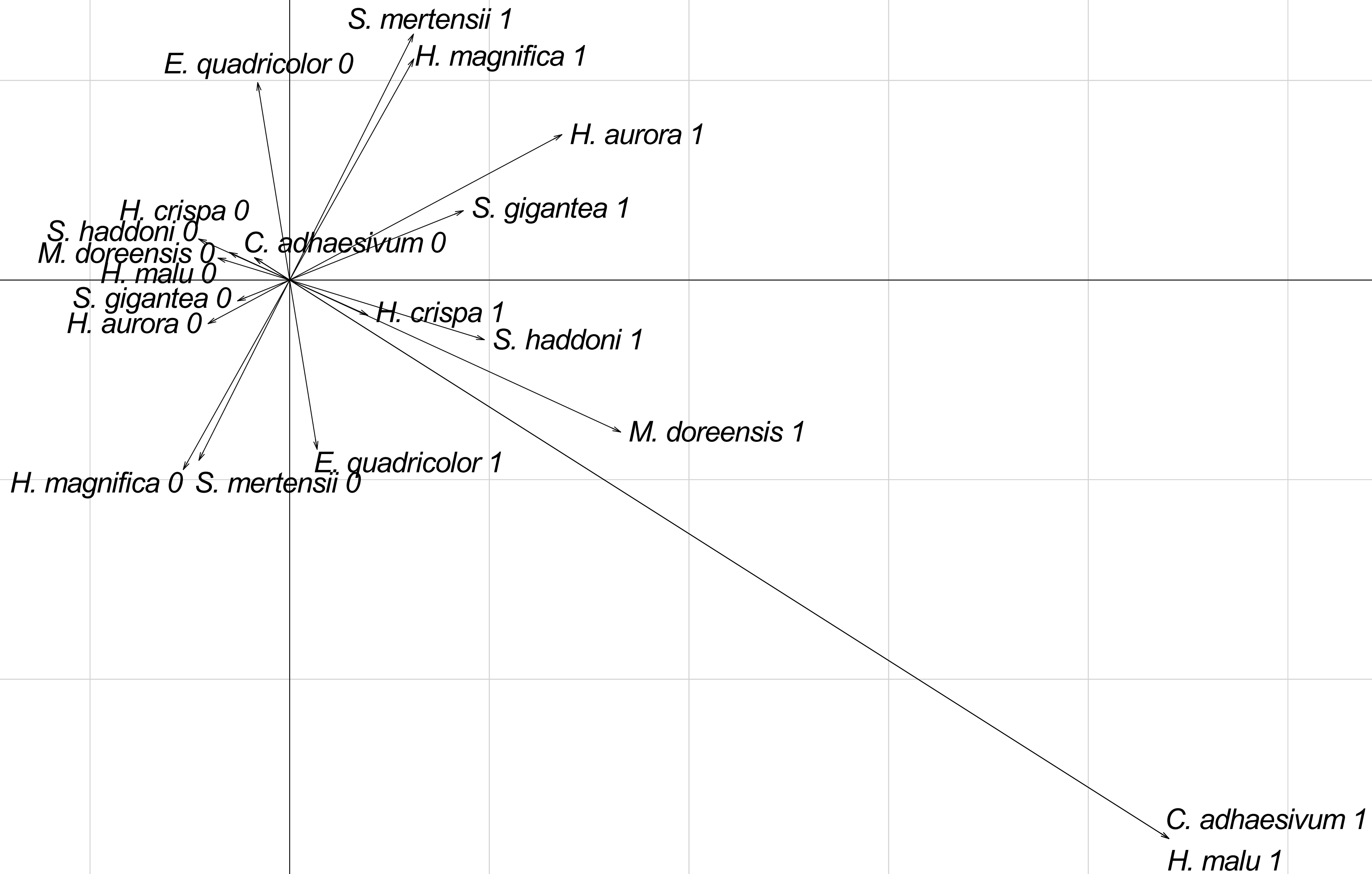

Supplement: Additional file 5 — Factorial map of the MCA analysis with eigenvectors for the axes 1 and 2. [file 1471-2148-12-212-S5.pdf]

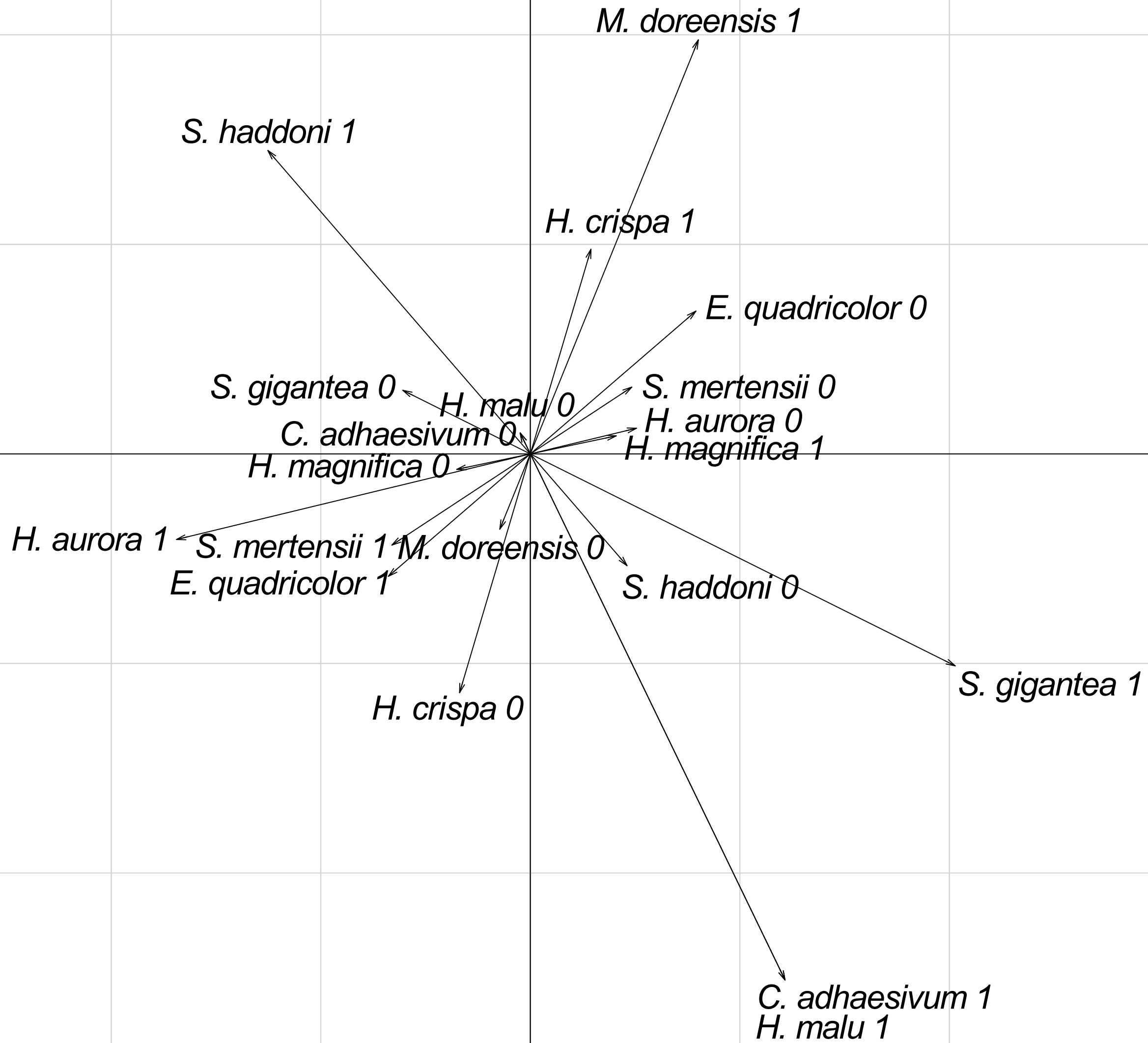

Supplement: Additional file 6 — Factorial map of the MCA analysis with eigenvectors for the axes 3 and 4. [file 1471-2148-12-212-S6.pdf]
